# Integrative analysis of differentially expressed genes and miRNAs predicts complex T3-mediated protective circuits in a rat model of cardiac ischemia reperfusion

**DOI:** 10.1038/s41598-018-32237-0

**Published:** 2018-09-14

**Authors:** Francesca Forini, Giuseppina Nicolini, Claudia Kusmic, Romina D’Aurizio, Milena Rizzo, Mario Baumgart, Marco Groth, Stefano Doccini, Giorgio Iervasi, Letizia Pitto

**Affiliations:** 1 Institute of Clinical Physiology, CNR, Pisa Italy, via G. Moruzzi 1, 56124 Pisa, Italy; 2 Laboratory for Integrative System Medicine (LISM), Institute of Informatics and Telematics (IIT), CNR, via G. Moruzzi 1, 56124 Pisa, Italy; 3 Leibniz Institute on Aging – Fritz Lipmann Institute (FLI), Jena, Germany; 4 Molecular Medicine, IRCCS Stella Maris, Pisa, Italy

## Abstract

Thyroid hormone (T3) dyshomeostasis in the cardiac ischemia-reperfusion (IR) setting negatively impacts on mitochondria function and extracellular matrix remodeling. The modulation of cardiac miRNAs may represent the underlying molecular mechanisms, but a systems biology perspective investigating this critical issue in depth is still lacking. A rat model of myocardial IR, with or without an early short-term T3-replacement, was used to predict putative T3-dependent miRNA-gene interactions targeted to mitochondria quality control and wound healing repair. As evidenced by mRNA and miRNA expression profiling, the T3 supplementation reverted the expression of 87 genes and 11 miRNAs that were dysregulated in the untreated group. *In silico* crossing and functional analysis of the T3-associated differentially expressed transcripts, identified a signature of interconnected miRNA-gene regulatory circuits that confer resistance to noxious cascades of acute stress. In this network the T3-down-regulated Tp53, Jun and Sp1 transcription factors emerge as critical nodes linking intrinsic cell death and oxidative stress pathways to adverse remodeling cascades. The data presented here provide a novel insight into the molecular basis of T3 cardioprotection in the early post-IR phase and highlight the contribution of a previously unappreciated complex T3-regulatory network that may be helpful in translating T3 replacement into clinical practice.

## Introduction

Heart disease following acute myocardial infarction represents a serious health problem and a major cause of death worldwide. Although timely reperfusion is mandatory to support cell function and to remove potentially noxious by-products of cellular metabolism, it also elicits pathogenetic processes that exacerbate injury due to ischemia *per se*^1,2^. Cardioprotective strategies targeting the activation of multiple pathologic pathways in the early stages of the post-IR wound healing process must be adopted to enhance cell resistance to death, to reduce adverse remodeling and to improve patient prognosis.

Thyroid hormones exert a fundamental role in cardiovascular system homeostasis^3^. Not surprisingly, even a small alteration of the thyroid function correlates positively with coronary risk factors and increased mortality for heart disease^4,5^. In patients with acute myocardial infarction (AMI) a condition of reduced T3 plasma level, known as low T3 state (lowT3S), represents a risk of cardiac disease progression and higher mortality^6,7^. Along this line, increasing clinical and experimental findings prompt for a cardioprotective role of lowT3S correction through T3 administration in cardiac disease^8,9^. However the clinical implementation of this therapeutic option is hampered by the residual concern of adverse effects. A comprehensive analysis of the cardioprotective networks triggered by T3 replacement in the context of post-IR signaling activation may help developing new therapeutic strategies and encourage pilot clinical trials.

In the complex scenario of IR, mitochondria quality control and extracellular matrix (ECM) homeostasis play a central role as major determinants of cardiac function^10,11^. On the one hand, mitochondrial dysfunction cause cell stress and cardiomyocyte death during both acute ischemic attack and post ischemic disease evolution^2,12^; on the other side, several profibrotic mediators, identified in the myocardium from the early steps of damage repair, shape the scar and determine heart failure development^11,13^.

Noteworthy, mitochondria and extracellular matrix have been previously suggested as main effectors of T3-mediated cardioprotection^14–16^. Although the underlying molecular bases are not completely understood, miRNA-based post transcriptional regulation has been proposed as a candidate mechanism to be explored in depth in animal models of cardiac IR^15,16^. Indeed, miRNA have been implicated in a wide range of physiological and pathological processes relevant to cardiac contractility, cell fate, stress response and myocardial fibrosis^17,18^. Since one single miRNA may affect the expression of several genes, as well as several miRNAs may target the same gene, to unravel the contribution of T3 to post ischemic signaling activation the integration of high throughput data is essential. To our knowledge such studies have not been conducted on pre-clinical models of cardiac IR.

Therefore, our objective was to identify putative T3-dependent miRNA-gene regulatory circuits targeted to noxious stimuli against cardiac mitochondria and interstitium in the post-IR setting. To this aim we applied a systems biology approach to integrate miRNA and mRNA expression profiling analysis in rats subjected to cardiac IR with or without post-IR T3 replacement.

## Results

### T3 replacement promotes post ischemic cardiac recovery without altering physiological parameters

We previously observed that about 30–40% of rats subjected to myocardial IR exhibited a phenomenon similar to the lowT3S observed in patients after AMI, i.e., an isolated reduction of serum T3 in the absence of overt thyroid disease. In these lowT3S rats we demonstrated that 48 h treatment (started 24 h after IR) with slightly supraphysiological dose of T3 (6 μg/kg/day) improved the post ischemic recovery of cardiac function at 3 days, although increasing heart rate^15,16^.

In the present study, to better mimic a physiological condition with a view to a translational perspective, we evaluated the cardioprotective effects of a new T3 dose that was sufficient to correct the post ischemic lowT3S without inducing other systemic changes, i.e. a T3 replacement dose. For this purpose, 24 h after induction of cardiac IR, rats were subjected to 48 h infusion with T3 at 3 μg/Kg day (IRT3 group) or its vehicle (IR group), functional and molecular results were compared with Sham operated rats used as control.

To validate our model as a preclinical study, we first needed to assess the safety and efficacy of the replacement dose. Sham, IR and IRT3 groups had similar plasma levels of free thriiodothyronine (FT3) at baseline (2.63 ± 0.22 pg/ml, 2.52 ± 0.21 pg/ml, 2.59 ± 0.24 pg/ml respectively). Consistent with the human disease, at 3 days post-IR, only the IR rats showed a significant reduction of FT3 indicative of lowT3S, while the IRT3 group maintained FT3 level comparable to those of Sham rats (Table 1). Moreover, T3 supplementation did not result in alteration of body weight, heart rate, respiration rate or body temperature that remained comparable among groups (Table 1). To assess the effect of the T3 replacement dose on heart function, we have evaluated parameters of heart geometry and contractile performance in diastole and systole by ultrasound imaging 3days after IR. As summarized in Table 1 no differences among group were observed in left ventricle (LV) end diastolic diameter or in the ratio of early to late ventricular filling velocities (E/A ratio). Instead T3 replacement resulted in better preserved LV end systolic diameter, ejection fraction and fractional shortening (Table 1). Taken together, these data attest the safety of the T3 replacement strategy and its effectiveness on the recovery of the post-ischemic LV contractile performance.

**Table 1:** Measurement of physiological and heart function parameters at 3 days post IR.

	Sham	IR	IRT3
***Physiological measurements***			
FT3 (pg/ml)	2.65 ± 0.34	1.78 ± 0.20*	2.91 ± 0.21
Body weight (g)	412 ± 53	400 ± 30	391 ± 23
HR (bpm)	378 ± 17	350 ± 35	376 ± 22
RR (bpm)	74 ± 4	80 ± 7	87 ± 11
T (°C)	36.3 ± 0.7	35.9 ± 0.6	36.4 ± 1.1
***Heart function***			
LVEDd (mm)	6.3 ± 0.3	6.8 ± 0.5	6.2 ± 0.9
LVESd (mm)	3.0 ± 0.2	4.1 ± 0.3**	3.0 ± 0.6
EF (%)	78 ± 5	62 ± 7**	76 ± 6
FS (%)	50 ± 3	42 ± 3*	52 ± 6
E/A	1.2 ± 0.6	1.5 ± 0.3	1.3 ± 0.1

Data are mean ± SD. LVEDd, left ventricular end-diastole diameter; LVESd, left ventricular end-systole diameter; EF: ejection fraction; FS: fractional shortening; E/A: E/A ratio; HR: heart rate; RR: respiration rate; T: body temperature. n = 5 animals per group *p < 0.02 and **p ≤ 0.006 vs Sham and IRT3.

### T3 replacement associates with rescue of the post ischemic gene transcriptional changes

Cardiac mitochondria and interstitium remodeling seem emerging targets of T3 mediated cardioprotection^15,16^. Here we analyze the expression of two focused panels of genes involved in the diverse cellular functions of mitochondrial biology and fibrosis signaling at 3 days after IR to gain a more thoroughly insight into the molecular effects of T3 early replacement on mitochondrial quality control and ECM homeostasis (see Supplementary Table S1 for gene list). With respect to Sham, the IR rats showed altered expression of 87 transcripts out of 150 total genes that gave an evaluable signal in the mitochondria profiler array (MPA) and fibrosis profiler array (FPA) (Tables 2 and 3). This post-IR expression signature was characterized by 18 up-regulated and 19 down-regulated genes within the MPA (Table 2), and 50 up-regulated genes within the FPA (Table 3). As shown in Table 2, the up-regulated transcripts within the MPA includes genes of the intrinsic apoptosis pathway, regulators of mitochondrial membrane polarization and potential, as well as mediators of mitochondrial metabolites and small solute import. The IR down-regulated transcripts of the MPA includes genes essential for mitochondrial replication and function as well as for the transport of metabolite and ions (Table 2). The up-regulated transcripts of the FPA comprise genes encoding ECM remodeling enzymes, Tgfb signaling molecules and inflammatory cytokines, along with additional genes pivotal in fibrosis (Table 3). The high amount of IR dysregulated genes, widespread along all the functional groups covered by the MPA and FPA, demonstrates that a broad pattern of gene expression relevant to both mitochondrial function and wound healing processes is altered under a post-IR lowT3S.

**Table 2:** Differentially expressed genes (DE-genes) within the mitochondria array (MPA) at 3 days post IR in the presence and absence of T3 replacement.

Gene name	EntrezID	Sham	IR	IRT3
***Apoptosis, mitochondrial membrane polarization and potential***				
Bak1	116502	0.92 (0.39)	4.6 (1.5)*	1.75 (0.49)*^#^
Bbc3 (Puma)	317673	0.99 (0.19)	2.28 (0.48)*	1.47 (0.3)*^#^
Bid	64625	0.94 (0.52)	15 (5.1)*	7.1 (2.55)*^#^
Bnip3	84480	1.05 (0.36)	0.43 (0.22)*	0.94 (0.18)^#^
Cdkn2a	25163	0.95 (0.72)	20.9 (4.6)*	14.4 (4.2)*^#^
Gclc	25283	0.97 (0.82)	4.2 (1.35)*	2.09 (0.65)*^#^
Pmaip1 (noxa)	492821	0.92 (0.54)	2.29 (0.59)*	1.37 (0.39)^#^
Sfn	313017	1.06 (0.91)	2.8 (1.39)*	1.02 (0.21)^#^
Tp53	24842	0.92 (0.29)	2.85 (0.13)*	2.0 (0.54)*^#^
***Mitochondrial transport of metabolites and small molecule solute***				
Cpt1b	25756	1.03 (0.61)	0.39 (0.18)*	1.04 (0.29)^#^
Cpt2	25413	1.05 (0.28)	0.43 (0.16)*	0.95 (0.46)^#^
Tspo	24230	1.06 (0.48)	1.69 (0.36)*	2.04 (0.54)*
Slc25a10	170943	1.19 (0.92)	2.1 (0.44)*	0.97 (0.35)^#^
Slc25a12	362145	1.13 (0.36)	0.49 (0.17)*	0.88 (0.33)^#^
Slc25a13	362322	1.18 (0.25)	0.55 (0.08)*	0.88 (0.1)^#^
Slc25a16	361836	1.0 (0.22)	2.03 (0.27)*	1.45 (0.14)*^#^
Slc25a17	300083	1.12 (0.22)	2.78 (0.41)*	2.5 (0.66)*
Slc25a20	117035	1.04 (0.34)	0.39 (0.13)*	0.76 (0.14)^#^
Slc25a21	171151	1.2 (0.44)	0.26 (0.21)*	0.27 (0.14)*
Slc25a24	310791	1.08 (0.62)	10.2 (2.58)*	5.37 (1.71)*^#^
Slc25a25	246771	0.93 (0.83)	0.3 (0.12)*	0.44 (0.14)*
Slc25a3	245959	1.15 (0.36)	0.38 (0.09)*	0.79 (0.3)^#^
Slc25a37	306000	1.03 (0.74)	2.27 (0.09)*	1.58 (0.2)*^#^
***Mitochondrial import and protein targeting to mitochondria***				
Cln8	306619	1.08 (0.73)	3.06 (1.23)*	1.96(0.34)*^#^
Hsp90aa1	299331	1.02 (0.36)	2.18 (0.44)*	1.76 (0.29)*
Mtx2	288150	0.97 (0.18)	0.63 (0.12)*	0.96 (0.22)^#^
Timm10	64464	1.04 (0.38)	0.47 (0.09)*	0.53 (0.07)*
Timm17a	54311	1.18 (0.25)	0.4 (0.11)*	0.65 (0.11)*^#^
Timm8b	64372	1.07 (0.21)	0.42 (0.18)*	0.64 (0.13)*^#^
***Mitochondrial replication, function, morphology and distribution***				
Ppargc1a (Pgc1α)	83516	1.05 (0.34)	0.26 (0.14)*	0.73 (0.24)^#^
Mfn1	192647	1.07 (0.42)	0.55 (0.09)*	0.92 (0.14)^#^
Mfn2	64476	1.05 (0.61)	0.49 (0.17)*	0.9 (0.42)^#^
Msto1	295237	1.14 (0.31)	2.75 (0.63)*	0.96 (0.51)^#^
Rnf135	303350	0.92 (0.67)	3.93 (1.3)*	1.7 (0.46)*^#^
Sod1	24786	1 (0.23)	0.6 (0.08)*	0.94 (0.17)^#^
Sod2	24787	1.17 (0.33)	0.7 (0.15)*	1.24 (0.55)^#^
Ucp4	85262	1.09 (0.49)	2.53 (1.05)*	1.01 (0.34)^#^

Genes are grouped according to the functional category provided by the array manufacturer. Data are median fold change relative to Sham and interquartile range (IQR, in brackets). *p < 0.017 vs sham, ^#^p < 0.01 vs IR.

**Table 3:** Differentially expressed genes (DE-genes) within the fibrosis array (FPA) at 3 days post IR in the presence and absence of T3 replacement.

Gene name	EntrezID	Sham	IR	IRT3
***ECM Components and ECM remodeling enzyme***				
Col1a2	84352	0.77 (0.23)	15.8 (0.48)*	8.60 (4.0)*^#^
Col3a1	84032	0.85 (0.22)	15.7 (3.5)*	8.26 (4.60)*^#^
Mmp2	81686	0.76 (0.50)	6.42 (1.23)*	1.84 (0.96)*^#^
Mmp8	63849	0.38 (0.66)	5.82 (2.24)*	2.62 (1.90)*^#^
Mmp9	81687	0.98 (0.90)	5.99 (7.26)*	5.4 (3.15)*
Mmp14	81707	0.56 (0.40)	16.1 (9.21)*	3.12 (0.24)*^#^
Plat	25692	0.58 (0.22)	4.51 (2.47)*	1.73 (0.83)*^#^
Serpine1	24617	1.00 (0.75)	12.0 (1.83)*	7.40 (3.67)*^#^
Serpin h1	29345	0.78 (0.41)	8.33 (7.04)*	6.78 (5.75)*
Timp1	116510	0.34 (0.51)	17.10 (10.95)	13.0(14.54)
Timp2	29543	0.82 (0.35)	4.42 (1.61)*	2.18 (0.79)*^#^
***Cellular Adhesion Inflammatory Cytokines and Chemokines***				
Ccl11	29397	1.00 (0.76)	2.62 (0.52)*	1.98 (1.89)*
Ccl12	287562	0.88 (0.89)	17.83 (9.11)*	18.01 (24)*
Ccl3	25542	0.86 (0.30)	16.0 (9.25)*	10.69 (4.85)*
Itgav	296456	0.90 (0.73)	4.32 (0.44)*	1.39 (0.38)*^#^
Itgb1	24511	0.93 (0.37)	6.10 (2.10)*	2.81 (0.75)*^#^
Itgb3	29302	0.94 (0.30)	5.06 (2.02)*	2.56 (0.89)^#^
Itgb5	257645	1.00 (0.54)	2.34 (0.89)*	1.28 (1.01)^#^
Cxcr4	60628	0.82 (0.62)	6.10 (4.6)*	1.54 (1.26)^#^
Il1b	16176	0.78 (0.23)	3.21 (1.15)*	2.19 (0.86)*
Il10	25325	0.78 (0.48)	4.9 (0.44)*	10.61 (8.87)*^#^
Ilk	170922	0.88 (0.61)	5.98 (2.11)*	2.37 (1.26)*^#^
Tnf	24835	0.78 (0.23)	3.2 (1.15)*	2.19 (0.86)*
Faslg	25385	0.93 (0.47)	3.20 (0.46)*	1.36 (0.42)^#^
***Growth Factors and Tgfb Superfamily***				
Bmp7	85272	0.78 (0.71)	2.22 (0.66)*	0.6 (0.23)^#^
Ctgf	64032	0.77 (0.24)	7.04 (2.54)*	3.0 (1.99)*^#^
Dcn	29139	0.83 (0.43)	2.25 (0.54)*	1.95 (0.34)*
Edn1	24323	0.80 (0.58)	4.05 (1.28)*	3.35 (1.02)*
Hgf	24446	0.94 (0.55)	9.64 (4.54)*	3.75 (1.57)*^#^
Ltbp1	59107	1.04 (0.62)	3.25 (0.62)*	1.75 (0.78)^#^
Smad2	29357	0.84 (0.55)	3.01 (0.74)*	1.60 (0.56)*^#^
Smad4	50554	0.79 (0.50)	2.80 (1.15)*	1.50 (0.89)
Smad6	367100	0.75 (0.43)	1.68 (0.50)*	1.00 (0.62)^#^
Smad7	81516	0.69 (0.49)	2.05 (0.92)*	1.56 (1.30)*
Tgfb r1	29591	0.62 (0.59)	10.7 (2.98)*	1.94 (0.64)*^#^
Tgfb r2	81810	0.55 (0.31)	4.18 (1.82)*	1.10 (0.63)*^#^
Tgfb1	59086	0.81 (0.56)	10.8 (4.75)*	3.31 (0.65)*^#^
Tgfb2	81809	0.78 (0.67)	14.4 (11.6)*	3.04 (2.86)*^#^
Tgfb3	25717	0.66 (0.91)	7.50 (4.96)*	1.18 (0.89)^#^
Tgif1	316742	0.85 (0.29)	2.85 (2.00)*	4.82 (0.59)*^#^
Thbs1	445442	0.74 (0.69)	24.75 (10.9)*	13.1 (4.14)*^#^
Thbs2	292406	0.91 (0.21)	9.89 (3.62)*	4.83 (4.27)*^#^
***Trascription factor and Epithelial to Mesenchimal Transition***				
Nfkb1	81736	0.83 (0.51)	3.21 (0.59)*	1.50 (0.38)*^#^
Snai1	116490	0.68 (0.53)	19.33 (6.16)*	9.80 (2.30)*^#^
Sp1	24790	0.87 (0.49)	3.00 (0.44)*	1.60 (0.47)*^#^
Stat1	25124	0.81 (0.52)	2.41 (0.75)*	1.27 (0.40)*^#^
Stat6	362896	0.72 (0.50)	6.67 (2.96)*	2.96 (1.54)*^#^
Jun	24516	0.72 (0.46)	2.54 (1.28)*	0.96 (0.86)^#^
Myc	24577	0.82 (0.39)	7.53 (4.53)*	7.57 (3.82)*

Genes are grouped according to the functional category provided by the array manufacturer. Data are median fold change relative to Sham and interquartile range (IQR, in brackets). *p < 0.017 vs sham, ^#^p < 0.01 vs IR.

**Figure 1:**
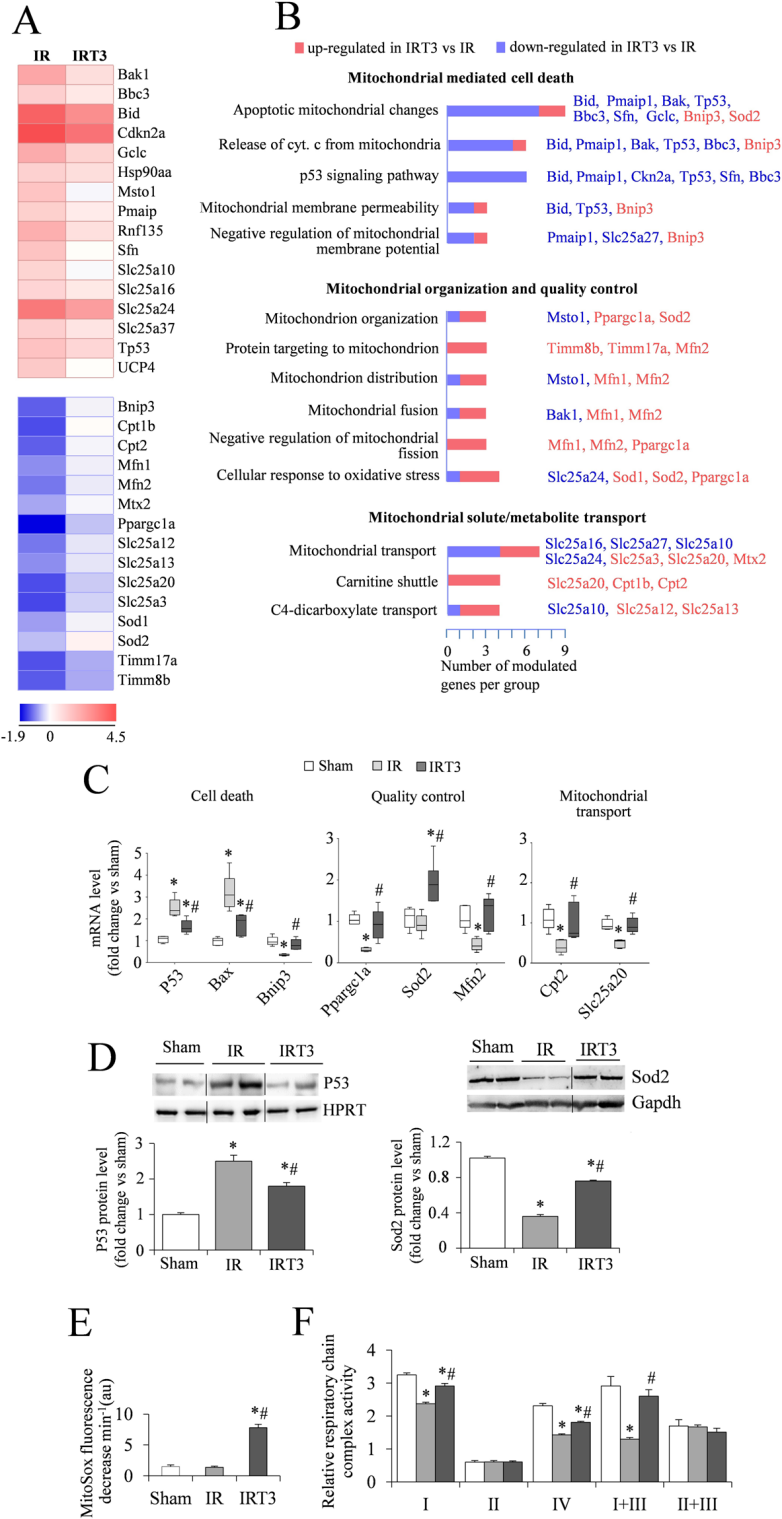
T3 replacement following IR induces gene transcriptional changes relevant to mitochondria organization and function. (**A**) Heat map examination of differentially expressed genes in the LV peri-infarcted area 3 d post-IR in the presence or absence of 48 h T3 replacement (columns) relative to Sham controls. Values (log_2_ of fold change) are shown by color and intensity of shading. Blue, down-regulated; red, up-regulated. *n* = 5 animals per group, *p* ≤ 0.016 IR vs IRT3. (**B**) Significantly enriched biological processes and pathways assessed by over-representation of gene ontology and KEGG terms using the T3 differentially expressed transcripts of the mitochondrial profiler array (MPA T3DE-genes) as input list. Blue, down-regulated by T3 versus IR; red, upreregulated by T3 versus IR (p < 0.05). (**C**) Box plot showing the validation of the array gene expression results in Sham, IR and IRT3 samples. Representative genes were selected according to the functional grouping and expression level was quantified by qRT-PCR. *n* = 5 animals per group. *p vs Sham ≤ 0.009, ^#^*p* vs IR ≤ 0.016. (**D)** Quantification of P53 and Sod2 protein level by western blot in Sham IR and IRT3 samples. *n* ≥ 3 animals per group. Upper panels: representative images, lower panel: data analysis *p vs Sham ≤ 0.016, ^#^*p* vs IR ≤ 0.016 (Full length images are reported in Supplementary Fig. S1 and S2). (**E**) Superoxide clearance measured in isolated mitochondria 3 d post-IR in the presence or absence of T3 replacement. *n* = 5 animals per group. *p vs Sham ≤ 0.0001, ^#^*p* vs IR < 0.0001. (**F**) Enzymatic activities of the respiratory chain (RC) complexes normalized to the citrate synthase activity. CI: NADH:ubiquinone reductase; CII: succinate:malonate dehydrogenase; CIV: cytochrome c oxidase; CI + CIII: NADH cytochrome c reductase; CII + CIII: succinate cytochrome c reductase; CS: citrate synthase *n* ≥ 3 animals per group. *p vs Sham ≤ 0.001, ^#^*p* vs IR ≤ 0.003.

**Figure 2:**
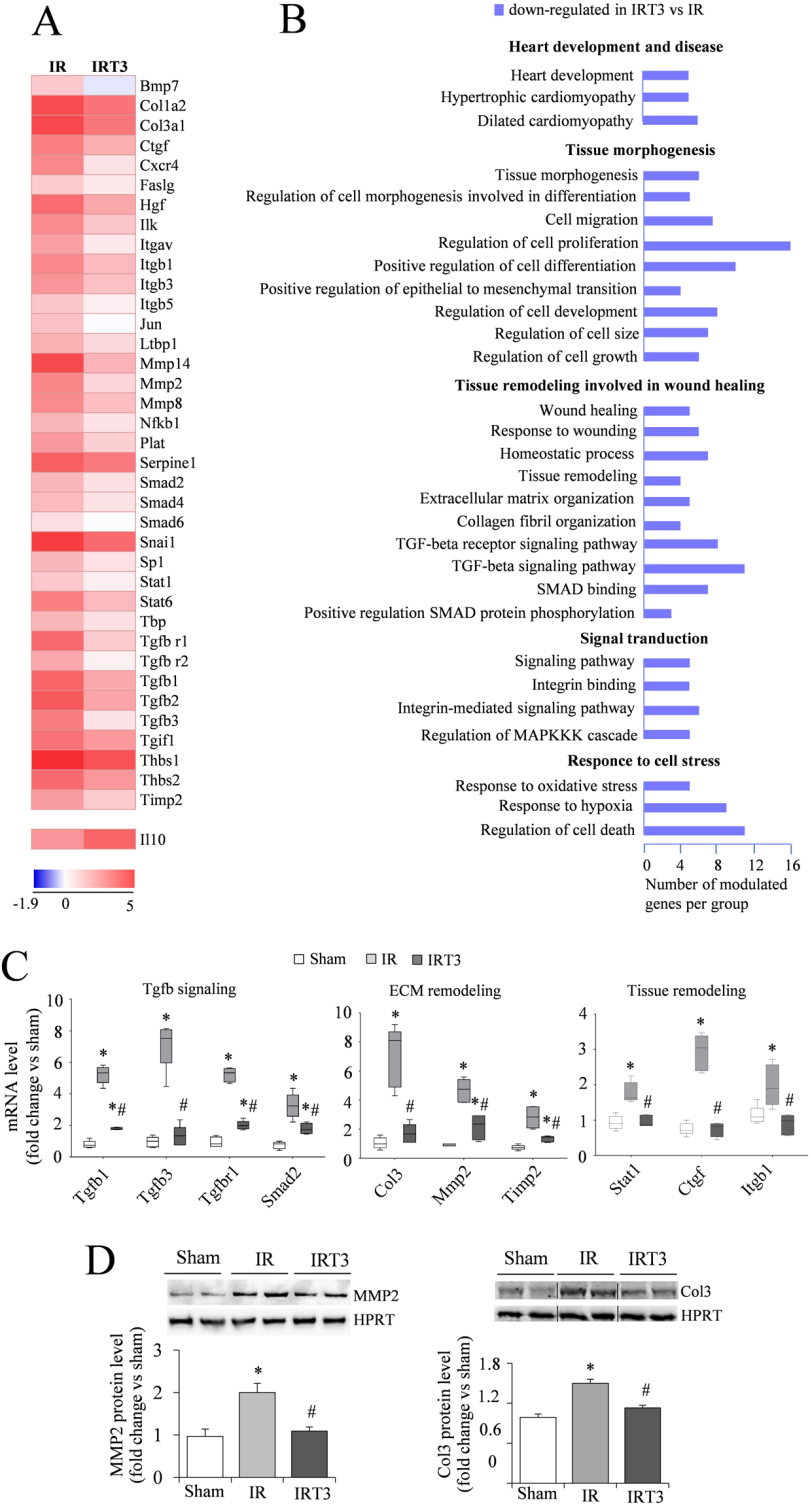
T3 replacement following IR induces gene transcriptional changes relevant to tissue remodeling and wound healing. (**A**) Heat map examination of differentially expressed genes in the LV peri infarcted area 3 d post-IR in the presence or absence of T3 replacement (columns) relative to Sham controls. Values (log_2_ of fold change) are shown by color and intensity of shading. Blue, down-regulated by T3 versus IR; red, up-reregulated by T3 versus IR. *n* = 5 animals per group, *p* ≤ 0.016 IR vs IRT3. (**B**) Significantly enriched biological processes and pathways assessed by over-representation of gene ontology and KEGG terms using the T3 differentially expressed transcripts of the fibrosis profiler array (FPA T3DE-genes) as input list. Blue, down-regulated by T3 versus IR **(**p < 0.05). (**C**) Box plot showing the validation of the array gene expression results in Sham, IR and IRT3 samples. Representative genes were selected according to the functional grouping and expression level was quantified by qRT-PCR. *n* = 5 animals per group. *p vs Sham ≤ 0.014, ^#^*p* vs IR ≤ 0.016. (**D**) Quantification of Mmp2 and Col3 protein level by western blot in Sham IR and IRT3 samples. *n* = 5 animals per group. Upper panels representative images, lower panels data analysis *p vs Sham = 0.004, ^#^*p* vs IR = 0.006 (Full length images are reported in Supplementary Fig. S3 and S4).

T3 replacement was associated to a significant differential modulation of 32 genes out of the 37 dysregulated transcripts within the MPA (Fig. 1A, Table 2), and 36 genes out of the 50 up-regulated transcripts within the FPA (Fig. 2A, Table 3), accounting for 86% and 72% of genes altered by the IR procedure in the MPA and FPA respectively. These results suggested that T3 replacement rescued the post-IR transcriptional changes.

### T3 replacement regulates the post ischemic myocardial gene expression signature to maintain mitochondrial function

To determine the biological and functional implications of these T3-dependent gene expression changes, we performed a functional enrichment analysis of the 32 plus 36 T3 differentially expressed genes (T3DE–genes) using DAVID annotation tool followed by data validation.

Within the MPA, the T3DE-genes were enriched into three main groups of biological processes implicated in (I) mitochondrial mediated cell death, (II) mitochondrial organization and quality control and (III) mitochondrial solute/metabolite transport (Fig. 1B). In line with the cardioprotective role of T3 replacement, almost all the genes involved in intrinsic apoptosis, p53-driven stress pathways, induction of mitochondrial depolarization and permeability transition were down-regulated by T3 (i.e Bak, Bid, Sfn, Pmaip1, Bbc3 and Tp53); while the T3 up-regulated genes were enriched in pathways that favor mitochondrial biogenesis and fusion (i.e. Pgc1α, Mfn1, Mfn2), antioxidant response to reactive oxygen species (i.e. Sod1 and Sod2), mitochondrial transport of metabolites necessary for ATP production and maintenance of mitochondrial membrane potential (i.e. Cpt1b, Cpt2; Slc25a12, Slc25a13) (Fig. 1B).

Data validation through qRT-PCR confirmed restored expression of relevant genes involved in p53-dependent proapoptotic cell death (Tp53, Bax, Bnip3), mitochondrial quality control (Ppargc1a, Sod2, Mfn2) and mitochondrial transport (Cpt2, Slc25a20) in the IRT3 group (Fig. 1C). To further support the array results, we showed that T3 infusion normalized the altered protein level of Tp53 and Sod2 that are key regulators of cell fate and superoxide detoxification (Fig. 1D). In line, we found an increased clearance of mitochondrial superoxide in the IRT3 group compared to IR (Fig. 1E). Globally, these findings suggest that T3 replacement is associated with activation of protective pathways that reduce cell death while enhancing mitochondrial quality control and function. Accordingly, spectrophotometric determination of oxidative phosphorilation (OxPhos) activities showed multiple defects of the mitochondrial respiratory chain in mitochondria isolated from myocardial tissue of IR rats compared to Sham, with significant reduction of CI (NADH:ubiquinone reductase) CI + CIII (NADH cytochrome c reductase) and CIV (cytochrome c oxidase) (Fig. 1E). A less impacted mitochondrial function was instead recorded in mitochondria from IRT3 group with a better preserved bioenergetics corroborating the protective role of T3 replacement (Fig. 1E).

### T3 replacement regulates the post ischemic myocardial gene expression signature to limit adverse remodeling

The T3DE-genes within the FPA were all down-regulated, with the exception of Il10 that was further up-regulated following T3 administration (Fig. 2B, Table 3). The enrichment analysis of this T3DE-gene set identified five main group of processes that orchestrate (I) heart development and disease, (II) tissue morphogenesis, (III) tissue remodeling involved in wound healing, (IV) signal transduction and (V) response to cell stress (Fig. 2B, Table 3). In detail the most relevant enriched pathways include: Tgfb signaling (i.e. Ltbp1, Tgfb1-3, Tgfb r1-2), Smad binding and signal transduction (i.e.Smad2, Smad4), integrin signaling (i.e Itgav, Itgb1), Mapk signaling (i.e Jun, Faslg), extracellular matrix organization (i.e Col1a2, Col3a1, Ctgf, Mmp2), morphogenesis and tissue remodeling (i.e. Ilk, Tgif, Snail, Bmp7) (Fig. 2B,). Data validation through qRT-PCR confirmed the reduced expression of relevant genes of the Tgfb signaling (Tgfb1, Tgfb3, Tgfb r1, Smad2), ECM remodeling (Col3, Mmp2, Timp2) and tissue remodeling, (Stat1, Ctgf, Itgb1) in the IRT3 rats compared to IR (Fig. 2C). Accordingly, the IRT3 group showed reduced protein levels of ECM remodeling effectors Mmp2 and Col3 with respect to IR (Fig. 2D). Taken together these data suggest that T3 replacement shapes the tissue repair processes by reducing the fibrotic reaction in the early phase of the post-IR wound healing.

### T3 administration associates with rescue of the post-ischemic miRNA expression profiling

**Figure 3:**
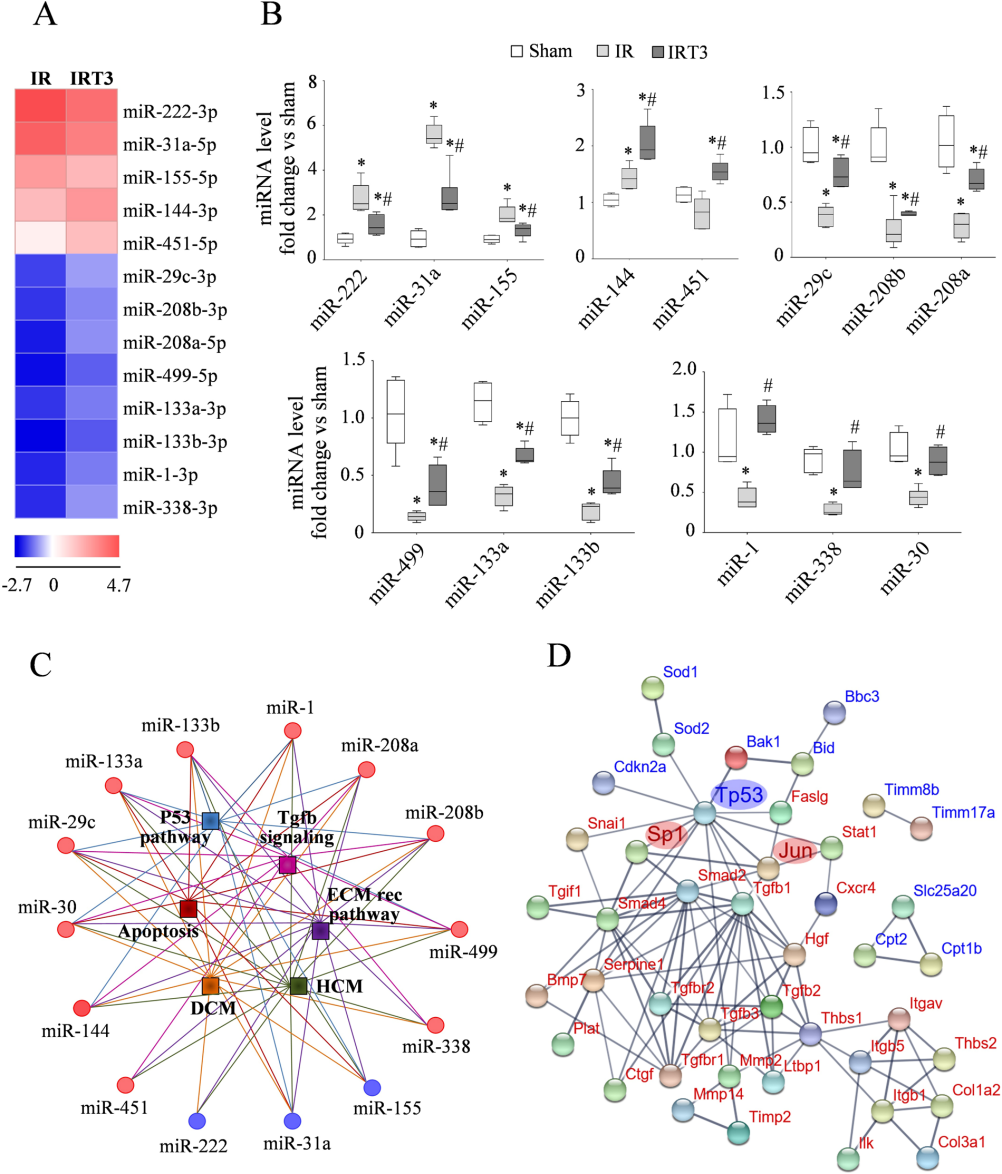
T3 administration following IR induces miRNA transcriptional changes relevant to cell survival, cardiac architecture and disease evolution. (**A**) Heat map examination of differentially expressed miRNAs in the LV peri-infarcted area 3 d post-IR in the presence or absence of T3 treatment (columns) relative to Sham controls. Samples used in this experiment were collected during a previous study using the same protocol but a T3 dose of 6 ug/Kg/day instesd of 3 ug/Kg/day^15^. Values (log_2_ of fold change) are shown by color and intensity of shading. Blue, down-regulated; red, up-reregulated. *p* < 0.05 IR vs IRT3. (**B)** Box plot showing the validation of the miRNA profiling by next generation sequencing in the new-dose-study samples. MiRNA expression level was quantified by qRT-PCR in the Sham, IR and IRT3 samples. *n* = 5 animals per group. *p vs Sham ≤ 0.014, ^#^*p* vs IR ≤ 0.016. (**C**) Enrichment analysis of the T3DE-miRNAs on the following pathways of interest: “apoptosis”, “p53 signaling”, “Tgfb signaling”, “extracellular matrix receptor pathway” (ECM rec patways), “hypertrophic cardiomyopathy” (HCM) and “dilated cardiomyopathy” (DCM). The lines connecting a miRNA to a pathway are depicted in the same color of the corresponding pathway symbol. Red circles, miRNAs up-regulated in IRT3vs IR, blue circles, miRNAs down-regulated in IRT3vs IR. p < 0.05. (**D**) Molecular interaction network of the T3DE-targets obtained using STRING and setting the minimum required score at 0.7. The transcripts of the MPA and FPA are displayed in blue and in red respectively. Line thickness indicates the strength of data to support the interactions. Disconnected nodes were omitted from the network display.

Recently we and others have suggested miRNAs as intriguing novel players in the multi-level system of T3-dependent cardioprotection^15,16,19,20^. Here we exploited the miRNA profiling by next generation sequencing (NGS) to systematically characterize the miRNAs involved in the beneficial cardiac effects of T3 administered after IR (see material and method for sample details). In the IR group we identified a total of 102 dysregulated miRNAs (42 down- and 60 up-regulated) that met the criteria of (I) a 2-fold up or down-regulation compared to Sham, (II) a mean of reads per million (rpm) higher than 100 and (III) an adjusted *p*-value < 0.05 (Supplementary Table S2). As shown in Fig. 3A, with respect to the IR group, T3 treatment significantly reverted the expression of 3 up-regulated miRNAs (miR-222, miR-31a and miR-155) and 8 down-regulated miRNAs (miR-1/133a cluster, miR-133b, myomiRs-208a/208b/499, miR-29c and miR-338) toward values observed in the Sham group. Notably, T3 administration increased the level of miR-144/451 cluster, the former was already up-regulated in the IR group with respect to the Sham while the latter showed similar level in IR and Sham groups (Fig. 3A). The results of NGS were confirmed by qRT-PCR analysis of all the differentially expressed miRNAs (Fig. 3B).

In accordance with previous reports^15,16^, several members of the miR-30 family were down-regulated by IR. In the presence of T3 replacement, all the miR-30 members showed a weaker down-regulation as compared to IR alone (see Supplementary Table S2) although at not significant level, which is probably due to the intrinsic difficulties in the detection by NGS of miRNAs whose sequences differ in only 1 or 2 bases. Even though miRNA-30 family was excluded from the list of IR vs IRT3 differentially expressed miRNAs (T3DE-miRNAs) shown in Fig. 3A, we decided to include it in the subsequent analysis as we had already reported a significant role of T3 in the up-regulation of miR-30 after IR in previous works^15,16^. In any case, the miR-30 up-regulation after T3 treatment was confirmed in the present study through qRT-PCR analysis (Fig. 3B).

### Integrative miRNA-mRNA analysis reveals the cardioprotective role of a complex T3 dependent network in the early post ischemia reperfusion setting

Next we aimed to evaluate whether the T3 differentially expressed miRNAs (T3DE-miRNAs) might be involved in the regulation of the biological processes emerged from the T3DE-genes functional enrichment analysis. To this purpose, we used MiRWalk to search for predicted target pathways of the T3DE-miRNAs restricting the analysis to the processes highlighted by the gene array profiling and that are critical for the evolution of the post ischemic cardiac disease evolution. As shown in Fig. 3C, and Supplementary Table S3, all the T3DE-miRNAs were predicted to have a role in the selected processes leading to adverse remodeling (“apoptosis”, “p53 signaling”, “Tgfb signaling” and “ECM receptor pathways”), and heart disease evolution (“hypertrophic cardiomyopathy” and “dilated cardiomyopathy”).

Given that the T3DE-miRNAs and the T3DE-genes share common pathways, we used MiRWalk to investigate whether the T3DE-genes were predicted targets of the T3DE-miRNAs restricting the analysis to the anti-modulated miRNA/mRNA couples. The list of the T3DE-miRNAs and their target mRNAs that display inverse modulation (T3DE-targets) is reported in Tables 4 and 5 according to the gene functional grouping. It is of note that the prediction algorithms identified miRNA potential recognition elements (MREs) within the vast majority of T3DE-genes of both MPA (31 out of 33 T3DE-genes) (Table 4) and FPA (31 out of 39 T3DE-genes) (Table 5). This integrative analysis of T3DE-genes and T3DE-miRNAs identified the presence of T3-modulated miRNA-gene regulatory circuitries that are predicted to counteract noxious pathways leading to adverse remodeling such as cell death, mitochondrial dysfunction, extracellular matrix disarray, inflammation and pro-fibrotic cascades (Tables 4 and 5). Interestingly, the vast majority of the T3DE-targets within the MPA and FPA share some common T3DE-miRNAs, with miR-133, miR-208 and miR-30 families showing the highest number of overlapping mRNAs (Tables 4 and 5).

**Table 4:** Anti-modulated T3DE-miRNAs and T3DE-targets from the mitochondria array (MPA) listed according to gene functional grouping.

Function	Down-regulated T3DE-targets	Up-regulated T3DE-miRNAs
***Intrinsic cell death***		
***Mitochondrial membrane depolarization and permeability***		
	Bak1	29c-3p; 133a-3p, 208a-5p; 208b-3p; 499-5p; 338-3p; 30-fam.
	Bbc3	29c-3p; 133a-3p, 1-3p; 208a-5p; 338-3p; 144-3p; 30-fam.
	Bid	29c-3p; 133a-3p, 133b-3p; 1-3p; 208a-5p; 208b-3p; 499-5p; 30-fam.
	Cdkn2a	133a-3p, 133b-3p; 208a-5p; 30-fam.
	Snf	29c-3p; 133a-3p, 133b-3p; 1-3p; 208a-5p; 208b-3p; 499-5p
	Tp53	133a-3p, 133b-3p; 1-3p; 208a-5p; 338-3p
	Slc25a27(UCP4)	29c-3p; 133a-3p, 133b-3p; 1-3p; 208a-5p; 208b-3p; 499-5p; 338-3p; 144-3p; 451-5p
	Bak1	29c-3p; 133a-3p, 208a-5p; 208b-3p; 499-5p; 338-3p
	Bbc3	29c-3p; 133a-3p, 1-3p; 208a-5p; 338-3p; 144-3p
***Mitochondrial substrate/solute transport***		
	Slc25a10	133a-3p; 1-3p; 208a-5p; 208b-5p; 338-3p
	Slc25a16	133a-3p, 133b-3p; 1-3p; 208a-5p; 208b-3p; 499-5p; 144-3p; 451-5p
	Slc25a24	29c-3p; 133a-3p, 133b-3p; 1-3p; 208a-5p; 208b-3p; 499-5p; 144-3p; 451-5p
	Slc25a37	133a-3p, 133b-3p; 1-3p; 208a-5p; 208b-3p; 499-5p; 338-3p; 144-3p; 451-5p
***Other***		
	Cln8	29c-3p; 133a-3p, 1-3p; 208a-5p; 208b-3p; 499-5p; 338-3p; 144-3p;0.451-5p; 30-fam.
	Gusb	29c-3p; 133a-3p, 1-3p; 208a-5p; 208b-3p; 499-5p; 338-3p; 144-3p; 451-5p; 30-fam.
	Msto1	133a-3p, 1-3p; 208a-5p; 499-5p; 144-3p; 30-fam.
	Rnf135	133a-3p, 133b-3p; 1-3p; 208a-5p; 208b-3p; 499-5p; 338-3p; 144-3p; 451-5p; 30-fam.
**Function**	**Up-regulated T3DE-targets**	**Down-regulated T3DE-miRNAs**
***Mitochondrial organization and quality control***		
	Ppargc1a	222-3p
	Mfn2	222-3p; 31a-5p; 155-5p
	Bnip3	222-3p; 31a-5p; 155-5p
	Sod1	222-3p
	Sod2	222-3p; 31a-5p; 155-5p
***Mitochondrial substrate/solute transport***		
	Cpt1b	222-3p
	Cpt2	222-3p; 31a-5p; 155-5p
	Mtx2	31a-5p
	Timm17a	222-3p; 31a-5p
	Timm8b	31a-5p; 155-5p
	Slc25a12	222-3p; 31a-5p; 155-5p
	Slc25a13	222-3p; 31a-5p; 155-5p
	Slc25a20	31a-5p
	Slc25a3	222-3p; 31a-5p; 155-5p

**Table 5:** Anti-modulated T3DE-miRNAs and T3DE-targets from the fibrosis array (FPA) listed according to gene functional grouping.

Function	Down-regulated T3DE-targets	Up-regulated T3DE-miRNAs
***ECM component and remodeling enzyme***		
*Epithelial to mesenchimal transition*	Col1a2	133a-3p, 133b-3p, 1-3p, 208a-5p; 144-3p; 30-fam.
	Col3a1	133a-3p, 133b-3p, 1-3p, 208b-5p; 144-3p, 499-5p; 30-fam.
	Ctgf	133a-3p, 133b-3p, 1-3p, 208a-5p, 208b-5p; 144-3p, 499-5p, 338-3p; 30-fam.
	Ilk	133a-3p, 133b-3p, 1-3p, 208a-5p, 338-3p; 30-fam.
	Mmp2	29c-3p; 133a-3p, 133b-3p; 1-3p; 208a-5p; 144-3p, 499-5p; 30-fam.
	Mmp14	29c-3p; 133a-3p, 133b-3p; 1-3p; 208a-5p; 144-3p; 30-fam.
	Plat	133a-3p, 133b-3p; 1-3p; 208a-5p; 208b-3p; 144-3p, 499-5p; 30-fam.
	Serpine1	133a-3p, 133b-3p; 1-3p; 208a-5p; 208b-3p; 338-3p, 144-3p, 451-5p,499-5p; 30-fam.
	Snai1	29c-3p, 133a-3p, 133b-3p; 1-3p; 208a-5p, 144-3p, 451-5p; 30-fam.
	Timp2	133a-3p, 133b-3p; 1-3p, 208a-5p; 338-3p; 30-fam.
***Cellular adesion***		
	Itgav	133a-3p, 133b-3p; 1-3p; 208a-5p; 144-3p; 30-fam.
	Itgb1	133a-3p, 133b-3p; 1-3p; 208a-5p; 208b-3p; 338-3p, 499-5p; 30-fam.
	Itgb5	29c-3p, 133a-3p, 133b-3p; 1-3p; 208a-5p; 208b-3p; 144-3p, 499-5p; 30-fam.
***Tgfb signal transduction***		
	Bmp7	133a-3p, 133b-3p, 1-3p; 208a-5p; 338-3p; 451-5p; 30-fam.
	Ltbp1	29c-3p, 133a-3p, 133b-3p; 1-3p; 208a-5p; 208b-3p; 338-3p, 144-3p, 499-5p; 30-fam.
	Smad2	133a-3p, 133b-3p; 1-3p; 208a-5p; 208b-3p; 338-3p, 144-3p, 499-5p; 30-fam.
	Smad4	133a-3p, 133b-3p; 1-3p; 208a-5p; 30-fam.
	Tgfb r1	29c-3p, 133a-3p, 133b-3p; 1-3p; 208a-5p; 208b-3p; 338-3p, 144-3p, 451-5p,499-5p; 30-fam.
	Tgfb r2	133a-3p, 133b-3p; 338-3p; 30-fam.
	Tgfb1	208a-5p; 30-fam.
	Tgfb2	29c-3p, 133a-3p, 133b-3p; 1-3p; 208a-5p; 208b-3p; 144-3p, 451-5p, 499-5p; 30-fam.
	Tgfb3	29c-3p, 133a-3p, 133b-3p; 1-3p; 208a-5p; 144-3p, 338-3p; 30-fam.
	Tgif1	133a-3p, 133b-3p; 1-3p; 208a-5p; 144-3p, 338-3p; 30-fam.
	Thbs1	133a-3p, 133b-3p; 208a-5p; 30-fam.
	Thbs2	133a-3p, 133b-3p; 1-3p; 208a-5p; 208b-3p; 144-3p, 338-3p, 451-5p, 499-5p; 30-fam.
***Transcription factors***		
	Jun	29c-3p, 133a-3p, 133b-3p; 1-3p; 208a-5p; 208b-3p; 338-3p, 144-3p, 451-5p,499-5p; 30-fam.
	Sp1	29c-3p, 133a-3p, 133b-3p; 1-3p; 208a-5p; 208b-3p; 338-3p, 451-5p,499-5p; 30-fam.
	Stat1	29c-3p, 133a-3p, 133b-3p; 1-3p; 208a-5p; 208b-3p; 338-3p, 144-3p, 451-5p, 499-5p; 30-fam.
***Inflammatory cytokine and chemokines***		
*Growth factors*	Cxcr4	133a-3p, 133b-3p, 1-3p, 208a-5p, 208b-5p; 144-3p, 499-5p, 338-3p, 451-3p; 30-fam.
	Faslg	133a-3p, 133b-3p, 1-3p, 208a-5p, 208b-5p; 144-3p, 499-5p, 338-3p, 451-3p; 30-fam.
	Hgf	133a-3p, 133b-3p, 1-3p; 144-3p; 30-fam.

Taken together the *in silico* findings suggest the presence of functional cross-talking among the T3DE-targets of the MPA and FPA. Therefore, we used the Search Tool for the Retrieval of Interacting Genes/Proteins (STRING) software to visualize the molecular and functional interaction among the T3DE-targets. As shown in Fig. 3D, the T3DE-targets form an integrated regulatory network where Tp53 emerges as an important interconnecting node linking the pro-apoptotic pathways of the MPA to the pro-fibrotic and adverse remodeling signaling of the FPA. This analysis also evidenced two separated minor networks involved in carnitine shuttle (Cpt1b, Cpt2 and Slc25a23) and protein targeting to mitochondrion (Timm8b, Timm17a) with nodes corresponding to the T3-up-regulated mRNAs that are predicted targets of the T3-down-regulated miRNAs.

## Discussion

Post ischemic cardiovascular disease, as well as cardioprotection, are multifactorial phenomena whose understanding requires systems-biology based approaches with all of the individual factors studied in relation with each other in an effort to unravel complex regulatory networks relevant to disease etiopathology and evolution. So far the beneficial cardiovascular effects of thyroid dyshomeostasis correction have been mainly inferred through the analysis of individual players in isolation^21,22^. This approach, although useful in unraveling several aspects of the T3-dependent cardiovascular effects, disregards the dynamic nature of the biological systems with all the synergistic or antagonistic actions of the involved factors.

The systems biology perspective adopted in this paper highlights for the first time the presence of integrated, T3-dependent miRNA-gene regulatory circuitries that are predicted to suppress cross-talking pathways involved in adverse cardiac remodeling and disease progression after an acute ischemic heart injury.

LV remodelling following acute IR is an early process in which mitochondrial disorganization, cell death, inflammatory response, fibroblast activation and ECM dyshomeostasis are the main myocardial triggers of adverse changes in tissue architecture leading to heart failure. As a consequence, an efficacious therapeutic strategy should counteract the initial activation of these detrimental pathways possibly without inducing adverse effects. In the present study we adopted a strategy of T3 replacement using a dose that resulted in effective restoration of FT3 plasma levels and preservation of mitochondrial function and cardiac performance without altering heart rate. We also verified that the new dose was appropriate to modulate the expression of well known TH responsive markers such as miR-208 and ppargc1 (pgc1α): the former is involved in the regulation of myosin isoform expression, the latter is a well-known regulator of mitochondrial biogenesis^14,23^. After checking safety and effectiveness of the new T3 dose, we assessed the role of the T3 replacement strategy in targeting the initial noxious processes induced by IR at the molecular level. To this purpose we analyzed the expression profile of gene arrays dedicated to mitochondrial quality control (MPA), wound healing and ECM homeostasis (FPA). As expected, IR procedure led to the up-regulation of processes involved in cardiomyocytes death and fibroblast activation including p53-dependent cascade, Mapk and Tgfb/Smad signaling. T3 supplementation was associated to the reversal of this expression signature. These findings extend and integrate previous data showing a protective role of TH against cardiomyocyte apoptosis and against the activation of p38/Mapk/Jnk signaling and pro-fibrotic cascade following heart ischemia^15,16,24,25^. In addition, we found that several transcripts of non-cardiomyocyte origin, implicated in inflammation and cell adhesion processes, were also modulated by T3 indicating that several different myocardial cell types may be involved in the protective effects of TH (see Fig. 2B). In support of this notion, previous independent works indicate beneficial actions of TH on fibroblasts, endothelial cells and vascular smooth muscle cells (reviewed in^26^).

Further, our gene functional enrichment analysis showed that many terms referring to heart disease condition, organ morphogenesis, regulation of cell size and growth were affected by T3 (Fig. 2B). Taken together our mRNA profiling data suggest that alterations in survival, proliferation and differentiation pathways, occurring in cardiomyocytes and non-cardiomyocyte cells during the first 3d post-IR and possibly involved in disease evolution, might be reverted by an early T3 replacement.

Such a degree of complexity suggests more sophisticated regulatory levels for TH signaling in addition to the classical control of gene expression through promoter binding. Recent evidence indicates an interplay between cardiac action of TH and the modulation of myocardial miRNAs suggesting that miRNA-dependent post translational modifications may have a critical role in TH signaling implicated in physiological cardiac hypertrophy, control of cardiomyocyte fate and regulation of tissue repair^15,16,19,20^. The present study reports for the first time a comprehensive miRNA profiling aimed to deepen our understanding of the cardiac T3 action following an acute IR event. Our results provide novel evidence that early post-IR T3 administration is paralleled by the modulation of a signature of cardiac miRNAs critically involved in the regulation of cardiac development, function and disease. Most of the miRNAs up-regulated by T3 have been previously shown to protect the infarcted myocardium by targeting cell death (miR-144/451 cluster, ref.^27^; miR-30a, ref.^15^; miR-133a, ref.^28^), mitochondrial dynamic (miR-499, ref.^29^) and profibrotic processes (miR-29, ref.^18^ miR-29, 133 and 30c ref.^16^). On the other side, the T3-down-regulated miR-31, -155 and -222, induced by IR, have been implicated in adverse cardiac remodeling and dysfunction during ischemic heart disease and heart failure^30–33^.

In addition, in the presence of T3 there was a tendency to a reduction of other stress responsive miRNAs such as miR-27 and miR-214 (Supplementary Table S2). Both in experimental models and in the clinical arena these miRNAs have been shown to evoke cardiac hypertrophy and failure by targeting the TH receptor beta1 and the TH inactivating enzyme deiodinase type III respectively^19,34,35^. These findings are in line with previous reports and confirm the critical role of miRNA modulation in TH signaling during stress conditions^19,34^.

In turn, alterations in the TH level have been shown to affect cardiac miRNA expression^36,37^. This reciprocal regulation between miRNAs and TH system strengthen the importance of maintaining the myocardial TH homeostasis to ensure a proper miRNA-dependent cardiac physiology. Our data indicate that a low dose T3 replacement strategy, sufficient to correct the post-IR lowT3S without altering plasma TH level, might be implemented in order to rescue both miRNA levels and cardiac function. In accordance with this interpretation, several lines of evidence suggest higher cardioprotective effects of physiological versus pharmacological T3 administration in the setting of cardiac disease^14,38,39^.

Our miRNA enrichment analysis on pathways emerged from the mRNA array experiments strongly suggests the causal involvement of miRNA modulation in the beneficial effect of T3 replacement. To further strengthen this association, the bioinformatic integration of the T3DE-miRNAs and anti-modulated T3DE-genes predicts a substantial contribution of the T3DE-miRNAs as direct regulators of the T3DE-gene expression profile observed in the post-IR setting. The number of overlapping T3DE-targets shared by the T3DE-miRNAs and the number of mRNAs bearing complementary sequences for more than one T3DE-miRNA suggest that an interplay between cooperativity and multiplicity of the T3DE-miRNAs may fine-tune gene expression to form complex regulatory networks within the heart tissue in the early phase of wound healing repair.

Accordingly, as a novel result, our *in silico* analysis evidenced that the T3DE-targets, form a network of cross talking nodes where the interactions of p53, c-JUN and Sp1 represent the connecting bridges between the mitochondria dependent cell death, adverse remodeling and pro-fibrotic cascade. These findings are consistent with a recent trascriptomic report where the gene expression signature associated to conditional heart-specific p53 knockout confers resistance to acute biomechanical stress and highlights the importance of p53 networks in the regulation of cardiac architecture, excitation-contraction coupling, mitochondrial biogenesis, and oxidative phosphorylation capacity^40^. Accordingly, we found that the miRNA-mRNA circuits associated to T3 replacement are predicted not only to inhibit the harmful pathways linked to p53 activation and adverse remodeling, but also to enhance protective processes that preserve mitochondrial function and integrity including carnitine shuttle, mitochondrial fusion, mitochondrial protein import and antioxidant activity. Studies like this might provide the basis to understand the molecular regulatory mechanisms modulated by miRNAs and thyroid hormone in cardiac IR and encourage to consider T3 replacement as a therapeutic choice in patients with acute ischemic cardiac disease and low T3S.

In conclusion, our data validate the effectiveness of a strategy of T3 replacement and support its clinical relevance. Also this work provides the first evidence that the beneficial cardiac effects of an early T3 replacement in the initial phase of the wound healing process is paralleled by the modulation of interacting molecules from different cell types putatively under the synergistic control of T3-responsive miRNAs. Although further studies are necessary to validate the predicted miRNA-gene circuitries, based on the present and previously published data, we envision a model in which stress stimuli affect the cardiac TH signaling leading to a reduction of T3 level; in this scenario the dysregulation of T3-dependent miRNAs and their target mRNAs favors cell death and adverse remodeling, which might be blunted by the maintenance of T3 cardiac homeostasis through T3 replacement.

## Materials and Methods

### Animal procedure

Animals used in this investigation conformed to the recommendations in the Guide for the Care and Use of Laboratory Animals published by the US National Institutes of Health (NIH Publication No. 85–23, revised 1996) and the protocol was approved by the Animal Care Committee of the Italian Ministry of Health (Endorsement n.552/20156-PR). All surgery was performed under anesthesia, and all efforts were made to minimize suffering. Myocardial infarction was produced by ligation of the left descending coronary artery of 12–15 weeks old adult male Wistar rats and weighing 310 ± 3 g. Rats were anesthetized using Zoletil® + xylazine (50 and 3 mg/kg respectively), connected to a respirator through an oropharyngeal cannula, and ventilated (Rodent Respirator 7025, Ugo Basile, Varese, Italy) with room air. A standard limb D1-D3 electrocardiogram (ECG) was continuously monitored and recorded at 2 kHz sampling rate (ML135 PowerLab/8SP equipped with ML135 Dual Bio Amp, ADI Instruments Ltd., Oxford, UK) using subcutaneous stainless steel electrodes (MLA0112 ECG Lead Switch Box, ADI Instruments Ltd., Oxford, UK). The heart was rapidly exposed through a thoracotomy at the left fourth intercostal space and pericardial incision. The left descendent coronary artery (LAD) was surrounded at about 2–3 mm from its origin by a 6–0 silk suture and its extremities were threaded through polyethylene tubing (PE-50) to form a snare for reversible artery occlusion. Ischemia was confirmed by ST segment elevation at ECG and visually assessed regional cardiac cyanosis. After 30 min of ischemia, the knot around the vessel was loosened and unrestrained reperfusion allowed. The silk suture was left *in situ*, the heart was returned to its normal position and the chest closed. A control group of rats underwent all surgical procedures except for the occlusion of the LAD (sham-operated group). Post-operatively, all rats were hydrated with physiological saline and given the analgesic buprenorphin 0.05 mg/kg s.c. (Temgesic®, Schering-Plough, Brussels, Belgium) before they gained consciousness.

### Experimental protocol

To mimic the low T3 syndrome observed in the clinical setting, only the IR rats which exhibited a >50% reduction of their basal serum FT3 level 24 h after surgery were randomly treated for 48 h with a constant subcutaneous infusion of 3 μg/kg/day T3 (IRT3, n = 5) or saline (IR, n = 5) via a miniosmotic pump (Alzet, model 2ML4, Palo Alto, CA, USA). Rats that at 24 h post IR exhibited serum FT3 reduction <50% of their basal value were excluded from the present study. To fix the T3 replacement dosage we used the plasma levels of free T3 (FT3) as a surrogate for the myocardial concentration value, due to the good correlation between the two indices previously demonstrated^15,41^. Hence, we set the T3 concentration in the osmotic pump to deliver subcutaneously 3 μg/kg/day, in order to maintain the plasma level of FT3 at about 3 pg/ml.

A group of sham-operated rats was treated with constant infusion of saline and used as control (Sham group, n = 5). Three days after surgery hearts were arrested in diastole by a lethal KCl injection. Cardiac tissue samples were obtained from the ischemic reperfused region and either stored at −80 °C until use or immediately processed for mitochondria isolation.

The samples used for miRNA profiling (one Sham, two IR, and two IRT3) were collected during a previous study in which the same model and T3 treatment protocol was used but a higher T3 dose (6 μg/kg/day vs 3 μg/kg/day of the present study)^15^. The new-dose-study samples were used for all the other experiments including the validation of miRNA profiling by qRT-PCR.

### Echocardiography study

All the animals underwent ultrasound (US) examination with a high-resolution imaging system (Vevo 2100, FUJIFILM VisualSonics Inc, Toronto, Canada) 3 days after infarction. A nose cone was used for maintaining animals under gaseous anaesthesia during the examinations (1.5% isoflurane in 1.2 L/min of pure oxygen) and heart rate (HR), respiration rate (RR) and body temperature (T) were monitored and acquired using the Advancing Physiological Monitoring Unit provided with the imaging station (Vevo Imaging Station, FUJIFILM VisualSonics Inc, Toronto, Canada). The thorax, previously shaved using depilatory cream (Nair, Church & Dwight Canada Corp., Mississauga, ON, Canada), was coated with acoustic coupling gel (SonoSite Cogel, Comedical Sas, Trento, Italy). For image acquisition a 20 MHz US probe (MS250, FUJIFILM VisualSonics Inc, Toronto, Canada), held in position by a mechanical arm, was employed. Images of the heart were acquired using B-mode modality in parasternal long axis (PLAX) and short axis (SAX) views, and then analyzed offline to assess cardiac structure and function. For each animal, transmitral inflow Pulsed-wave Doppler (PW-Doppler) images were obtained in apical 4-chamber view: the ratio of the early to late ventricular filling velocities (E/A) was calculated elaborating mitral inflow data and used as diastolic function parameter.

### Mitochondria isolation and functional analysis

Mitochondria were purified according to the manufacturer’s protocol (MITO-ISO1; Sigma) and as previously described (Forini). Cardiac tissue was homogenized in buffer containing 10 mM Hepes, 200 mM mannitol, 70 mM sucrose and 1 mM EGTA (PH 7.5) and centrifuged at 2000 *g* at 4 °C for 5 min. The supernatant was centrifuged at 11000 *g* at 4 °C for 20 min to collect the pellet. The mitochondria were suspended in storage buffer at pH 7.5 containing 10 mM HEPES, 250 mM sucrose, 1mMATP, 0.08 mM ADP, 5 mM sodium succinate, 2 mM K2HPO4 and 1 mM DTT and stored at −80 °C until use. An aliquot of the suspended pellet was assayed for protein content with the BioRad protein assay kit.

The enzymatic activities of RC complexes were assayed spectrophotometrically on intact mitochondria and expressed as a ratio to the activity of citrate synthase (CS), a mitochondrial matrix enzyme used to account for mitochondrial mass. Kinetic assays were performed at 30 °C using a Beckman Coulter DU760 spectrophotometer following standard methods already reported in Nesti C *et al*.^42^. Complex I (NADH:ubiquinone reductase) activity was measured by the rotenone sensitive oxidation of NADH at 340 nm. Complex II (succinate dehydrogenase) activity was measured by the malonate sensitive reduction of succinate at 600 nm. Complex I/III (NADH: cytochrome c reductase) activity was measured by NADH dependent reduction of cytochrome c at 550 nm. Complex II/III (succinate:cytochrome c reductase) activity was measured by succinate dependent reduction of cytochrome c at 550 nm. Complex IV (cytochrome c oxidase) activity was measured by the oxidation of reduced cytochrome c at 550 nm. CS activity was determined by the formation of 5-thio-2-nitrobenzoic acid at 412 nm.

For the assessment of mitochondrial superoxide clearance, mitochondria were incubated at 37 °C in medium containing 125 mM KCl, 2 mM K_2_HPO_4_, 20 mM HEPES, 5 mM pyruvate, 4 mM MgCl_2_, 3 mM ATP and 50 μM EGTA, pH 7.0. To increase mitochondrial superoxide production, 2 μM of the complex III inhibitor Antimycin-a was added to the reaction buffer. Five μM of the mitochondrial superoxide specific dye MitoSOX Red (Invitrogen) was added to a final volume of 200 μl before starting measurement. The rate of the fluorescence decay, indicating superoxide detoxification, was recorded at 510 nm excitation wavelength and 570 nm emission wavelength. The slope of the linear tract of the curve obtained in the first 18 min of the reaction was used as an index of the superoxide clearance rate. To account for aspecific signal, such as mitoSOX-reduction or fluorescence bleaching, a blank experiment was performed in mitochondria free sample containing only the mitochondria suspension buffer, no appreciable changes over time were recorded. All mitochondrial assays were performed in triplicate using the microplate reader Infinite M200 PRO (TECAN).

### Western blot analysis

The frozen cardiac tissue from the LV ischemia reperfused zone was pulverized and homogenized at 4 °C in lysis buffer (20 mM Tris-HCl, pH 8.0, 20 mM NaCl, 10% glycerol, 1% NP40, 10 mM Lethylenediaminetetraacetic acid (EDTA), 2 mM phenylmethylsulfonyl fluoride (PMSF), 2.5 μg/mL leupeptin, 2.5 μg/mL pepstatin) with the TissueLyser instrument (Qiagen). After centrifugation at 11.000 g for 15 min, the supernatant was collected and stored at −80 °C until use. Protein concentrations were determined by bicinchoninic acid assay (Pierce, Thermo Scientific). Equal amounts of proteins (30 μg) were separated on 4–12% polyacrylamide electrophoresis gel (Bolt Bis Tris mini gels, Life Technologies) and transferred to iBlot 0.2-mm polyvinylidene fluoride membranes (Life Technologies).

After blocking of the nonspecific binding sites, the membranes were incubated with specific primary antibodies for p53 (1:1000, Cell Signaling Technology), Sod2 (1:2000, Millipore), Mmp2 (1:1000, Cell Signaling Technology), Col3 (1:2000, Sigma-Aldrich), Gapdh (1:10000, Sigma-Aldrich) and HPRT (1:10000, ab 109021, Abcam). Subsequently, the membranes were incubated with secondary anti-rabbit or anti-mouse IgG conjugated with horseradish peroxidase (1:3000, Cell Signaling Technology). Protein signals were detected by Clarity ECL Substrate (Bio-Rad) according to the manufacturer’s instruction. Images were acquired with the integrated camera of the Alliance Mini2 Chemiluminescence Documentation System (UVITEC Cambridge). The resulting western blot bands were scanned using ALLIANCE-CAPT Advance software (UVITEC Cambridge). Band intensity was evaluated after excluding signal saturation and normalized using HPRT or GAPDH (see Supplementary Figs S1–S4 for additional information).

### RNA extraction and cDNA synthesis

Total RNA was extracted from homogenized heart tissue using the RNeasy mini kit (QIAGEN, Italy) following the manufacturers’ instructions. RNA quality and amount was determined using the Agilent Bioanalyzer 2100 and the RNA 6000 Nano Kit (Agilent Technologies) after a DNA digestion step with DNAse (invitrogen), to remove genomic DNA contamination. cDNA for quantification of gene and miRNA expression was generated in a single-step reaction from 1 μg of RNAs using the Miscript Reverse Transcription Kit (Biorad) or the miScript Reverse Transcription Kit (Qiagen) respectively, following the manufacturer’s instructions. Resulting cDNAs were stored frozen at −20 °C until assayed.

### PCR array profiling

The PCR-array gene expression profiling was performed using the Biorad PrimePCR plates dedicated to mitochondria gene panel (Mitochondria SAB Target List R96) and to fibrosis gene panel (Fibrosis SAB Target List R96) according to the manufacturer’s instruction. Expression levels were quantified by SYBR Green chemistry in 96 well plates with a Biorad CFX96 Real-Time System (Biorad) Five biological replicates were performed for each group of rats. Each PCR-array plate contained quality control wells for assaying RNA quality, reverse transcription quality, PCR quality and DNA contamination. Cq analysis was performed by the Bio-Rad CFX Manager software with a constant baseline adjustment of the relative fluorescent units for all array runs to provide for accurate comparisons across samples. Data were normalized against the geometric mean of Tbp, Gapdh and Hprt housekeeping genes using the ΔΔCq method.

### cDNA library preparation and next generation sequencing

Library preparation and sequencing was done using Illumina’s NGS platform as previously described^42,43^. The purified libraries were quantified on the Agilent DNA 7500 chip and subjected to sequencing on an Illumina HiSeq. 2500 in high-output, 50 bp single-read mode in pools of 9 per lane. Sequencing chemistry v3 was used. Read data were extracted in FastQ format using the Illumina supported tool bcl2fastq v1.8.3. Sequencing produced a mean of 26.2 million reads per sample (range 5.3M–37.3M) resulting in a mean of 14.7 million reads per sample (range 3.5M–18.7M) after trimming and quality filtering.

### MiRNA identification and differential expression analysis

The processing and annotation of small RNA-Seq raw data was performed using miRExpress (v2.1.3)^44^ to identify rattus pre-miRNAs present in mirBase repository (rel. 20) with at least 95% of sequence identity and a length tolerance range of 4 bp for mapping. Differential expression analysis was performed using Bioconductor’s package DESeq^45^. The reads count, used as measure of miRNAs quantification, was first normalized by library size factors to a common scale. p-values were estimated using a negative binomial distribution model and local regression after dispersion estimation in pooling mode. Raw p-values were finally adjusted for multiple testing using the Benjamini and Hochberg^46^ procedure controlling the false discovery rate (FDR). miRNAs with an adjusted p-value < 0.05 were considered to be differentially expressed.

### Quantitative real-time RT PCR

Both gene array results and NGS miRNA profiling data were validated by quantitative real-time reverse transcription PCR (qRT-PCR) as previously described^15,16^ using the new-dose-study samples (n = 5 animals per group). Genes and miRNA expression levels were quantified by SYBR Green chemistry with the Rotor-Gene Q 2-Plex (QIAGEN) and normalized respectively against Hprt or small nuclear RNA-U1 (snRNA-U1) and small nucleolar RNA, H/ACA Box 55 (snora55). The primer used in the qRT-PCR experiments are listed in Supplementary Table S4.

### Gene Ontology and pathways analysis

To gain more mechanistic insights, analyses of the overrepresented biological processes and KEGG pathways were conducted by using the functional annotation tool available within DAVID Website (https://david.ncifcrf.gov) and the lists of T3DE-genes as input.

### miRNA target prediction and analysis of molecular interaction network

The list of T3DE-miRNAs resulting from NGS was analyzed by the miRWalk2 tool (http://zmf.umm.uni-heidelberg.de/apps/zmf/mirwalk2/) to associate the T3DE-miRNAs to pathways of interest and to predict miRNA targets among the lists of T3DE-genes. Finally, to create an integrated network of interaction among the T3DEmRNAs, all the mRNAs potentially targeted by the differentially expressed miRNAs were ran in the Search Tool for the Retrieval of Interacting Genes/Proteins (STRING) database (https://string-db.org/) setting the minimum required interaction score at 0.700.

### Statistical analysis

Data were normalized to Sham pool. When conditions for parametric test were verified, differences among groups were evaluated with one way ANOVA, followed by Bonferroni post hoc correction; in these case data are expressed as mean ± SEM and differences were considered statistically significant at a value of p < 0.05. Data obtained from PCR-arrays and all the qRT-PCR quantifications were compared by means of the nonparametric Kruskall–Wallis test followed by a Mann–Whitney U-test (adjusting the α-level by Bonferroni inequality), in these cases data are express as median and interquartile range (IQR) and differences were considered statistically significant at a value of p < 0.017.

## Electronic supplementary material

Supplementary material

## Data Availability

All relevant data are within the paper and its supplementary information files, except for NGS data that have been deposited in NCBI’s Gene Expression Omnibus (Edgar *et al*., 2002) and are accessible through GEO Series accession number GSE116046 (https://www.ncbi.nlm.nih.gov/geo/query/acc.cgi?acc=GSE116046).
