# Supplementary material for: Integrative analysis of differentially expressed genes and miRNAs predicts complex T3-mediated protective circuits in a rat model of cardiac ischemia reperfusion
Source: Sci Rep. 2018 Sep 14;8:13870. doi: 10.1038/s41598-018-32237-0 (PMC6138681; doi:10.1038/s41598-018-32237-0)
Supplement: Supplementary file 1 — Supplementary material [file 41598_2018_32237_MOESM1_ESM.pdf]

Supplementary Information

Integrative analysis of differentially expressed genes and miRNAs predicts complex T3-mediated protective circuits in a rat model of cardiac ischemia reperfusion

*Forini Francesca 1, Nicolini Giuseppina 1, Kusmic Claudia 1, D'Aurizio Romina 2, Milena Rizzo 1, Baumgart Mario 3, Groth Marco 3, Doccini Stefano 4, Iervasi Giorgio1, Pitto Letizia 1

1 Institute of Clinical Physiology, CNR, Pisa Italy, via G. Moruzzi 1, 56124 Pisa, Italy

2 Laboratory for Integrative System Medicine (LISM), Institute of Informatics and Telematics (IIT), CNR, via G. Moruzzi 1, 56124 Pisa, Italy

3 Leibniz Institute on Aging – Fritz Lipmann Institute (FLI) Jena Germany

4. Molecular Medicine, IRCCS Stella Maris, Pisa, Italy

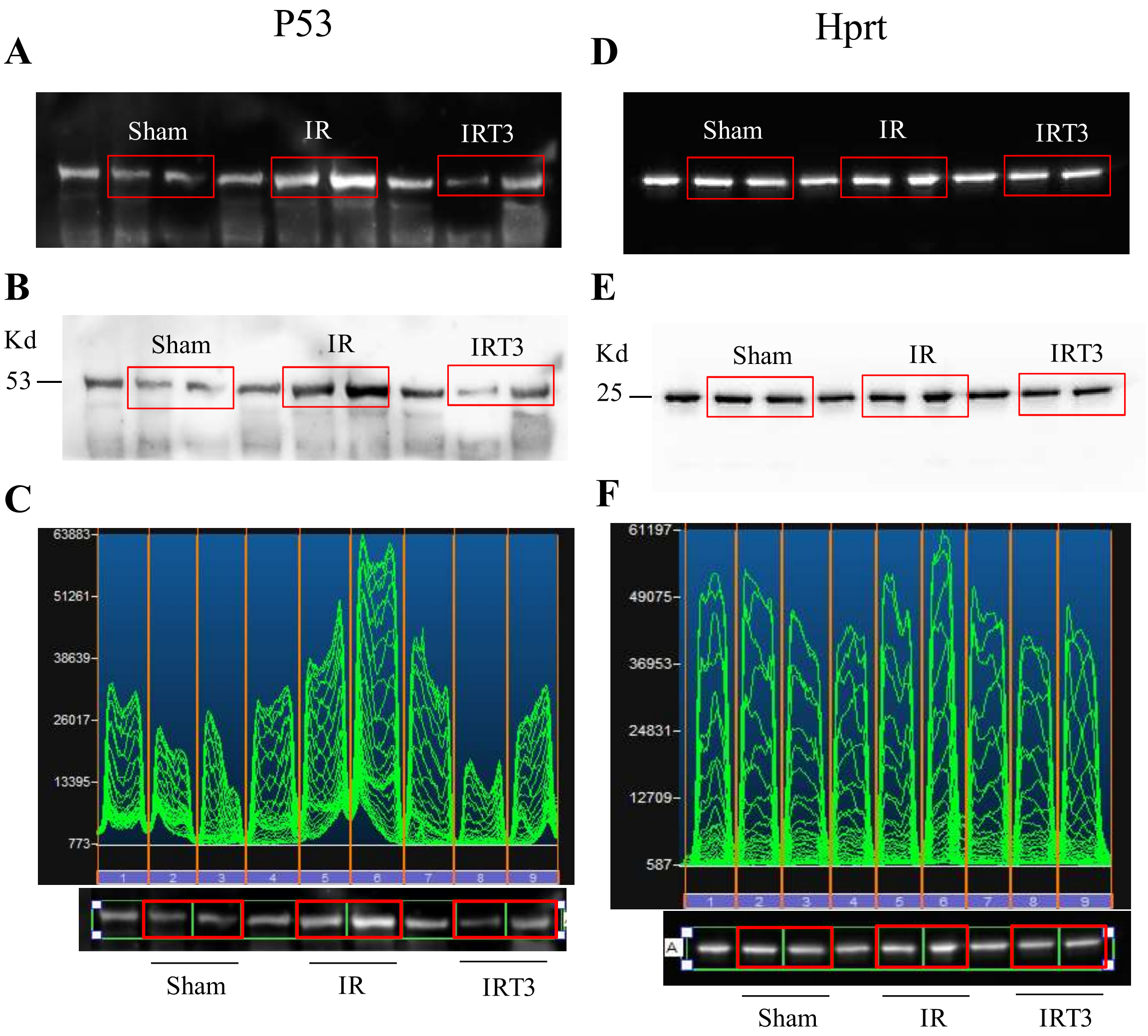

Supplementary Figure S1. P53 protein level evaluation. **A**. Unprocessed p53 specific chemiluminescent signal as acquired with the camera of the Alliance Mini2 Chemiluminescence Documentation System (UVITEC Cambridge). B. The original image was inverted for generating the cropped Figure 1D shown in the main text. C. The unprocessed original image was used to quantify the optical density through the ALLIANCE-CAPT Advance Software (UVITEC Cambridge) after excluding signal saturation as evidenced in the upper plot of the panel C. The bands of interest used in the cropped Figure 1D are marked with the red rectangles. C, D and E: Hprt specific chemiluminescent signal used for normalization was acquired and analyzed as described for p53.

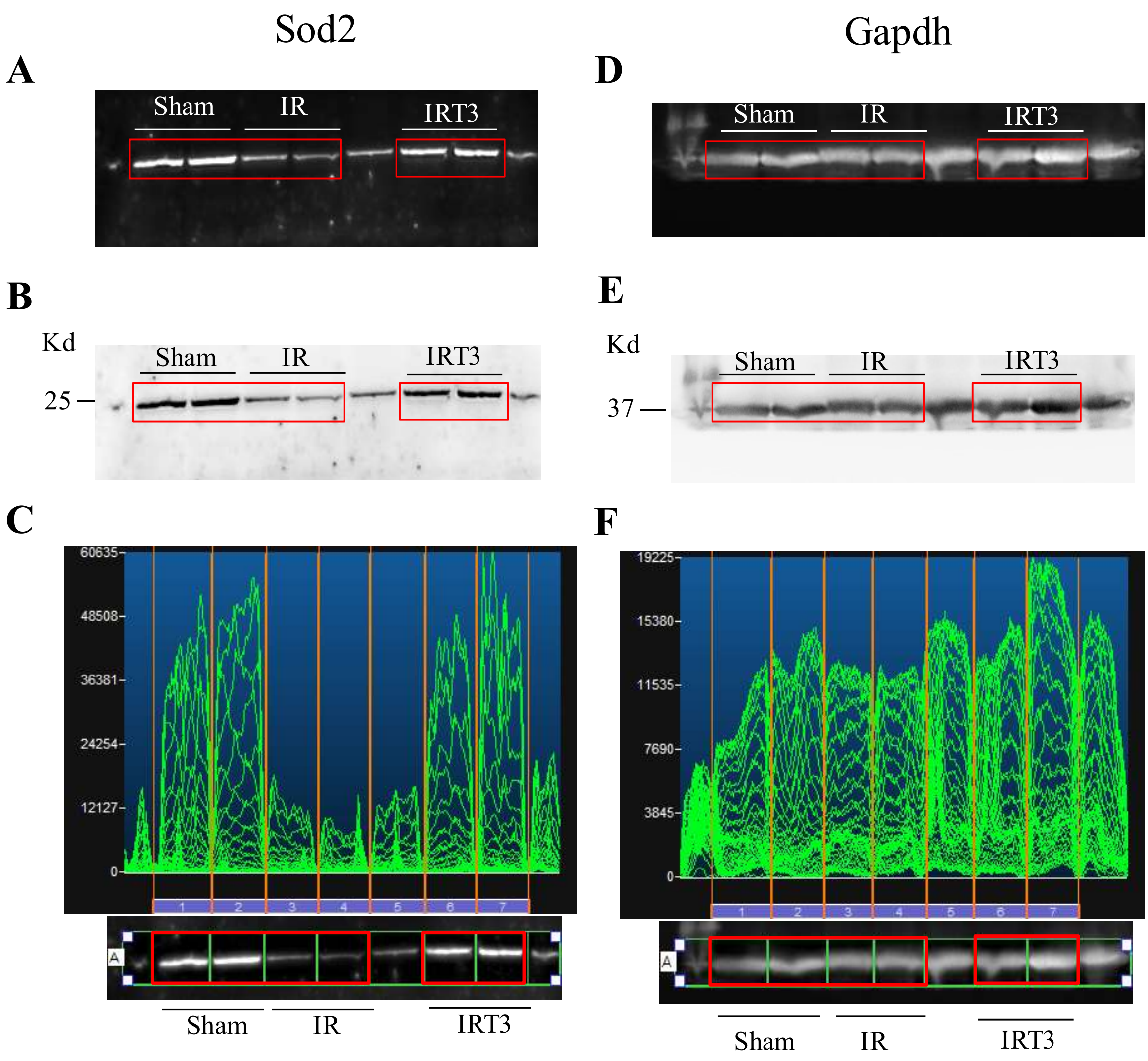

Supplementary Figure S2. Sod2 protein level evaluation. **A**. Unprocessed Sod2 specific chemiluminescent signal as acquired with the camera of the Alliance Mini2 Chemiluminescence Documentation System (UVITEC Cambridge). B. The original image was inverted for generating the cropped Figure 1D shown in the main text. C. The unprocessed original image was used to quantify the optical density through the ALLIANCE-CAPT Advance Software (UVITEC Cambridge) after excluding signal saturation as evidenced in the upper plot of the panel C. The bands of interest used in the cropped Figure 1D are marked with the red rectangles. C, D and E. Gapdh specific chemiluminescent signal used for normalization was acquired and analyzed as described for Sod2.

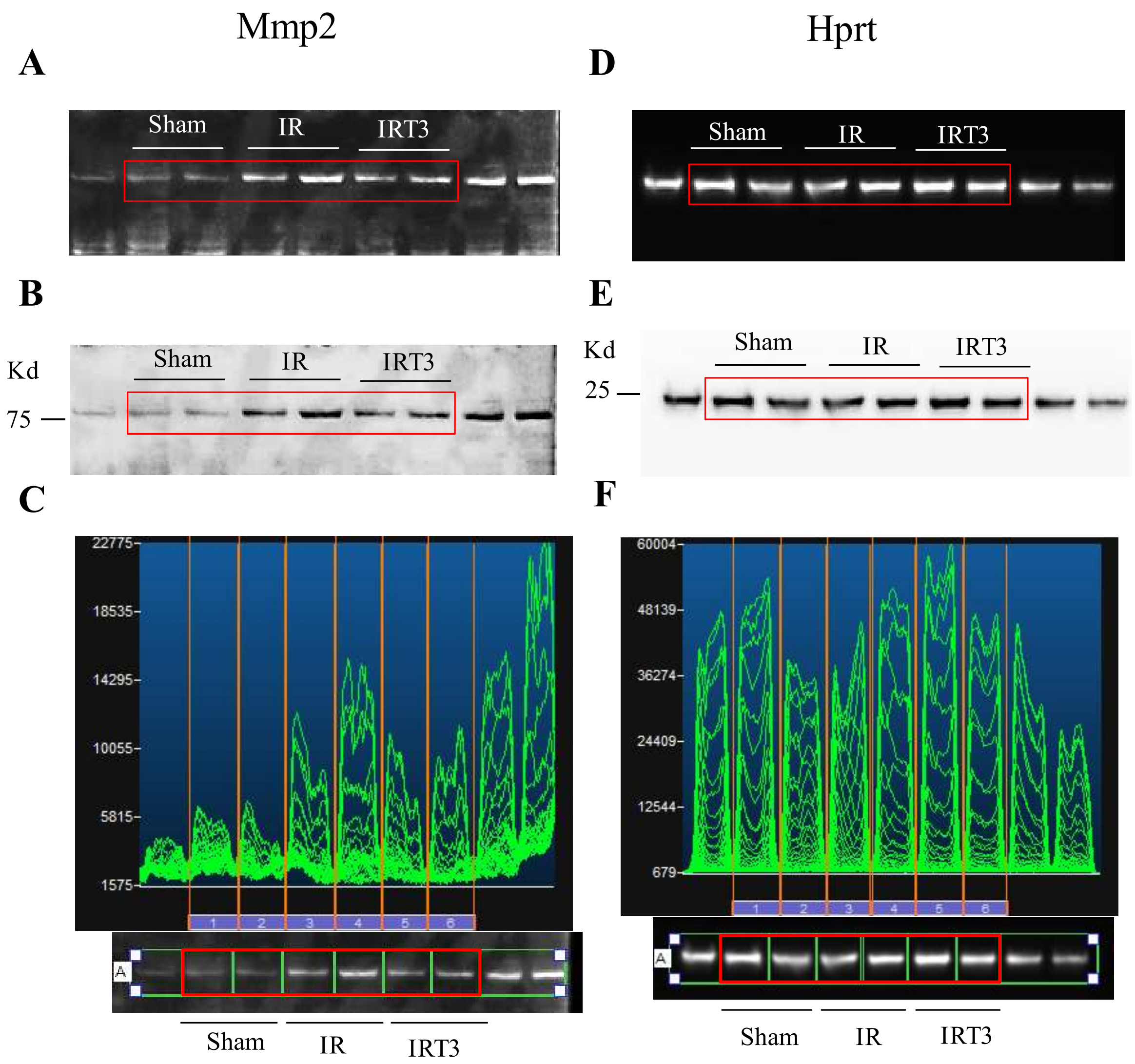

Supplementary Figure S3. Mmp2 protein level evaluation. **A.** Unprocessed Mmp2 specific chemiluminescent signal as acquired with the camera of the Alliance Mini2 Chemiluminescence Documentation System (UVITEC Cambridge). B. The original image was inverted for generating the cropped Figure 1D shown in the main text. C. The unprocessed original image was used to quantify the optical density through the ALLIANCE-CAPT Advance Software (UVITEC Cambridge) after excluding signal saturation as evidenced in the upper plot of the panel C. The bands of interest used in the cropped Figure 2D are marked with the red rectangles. C, D and E: Hprt specific chemiluminescent signal used for normalization was acquired and analyzed as described for Mmp2.

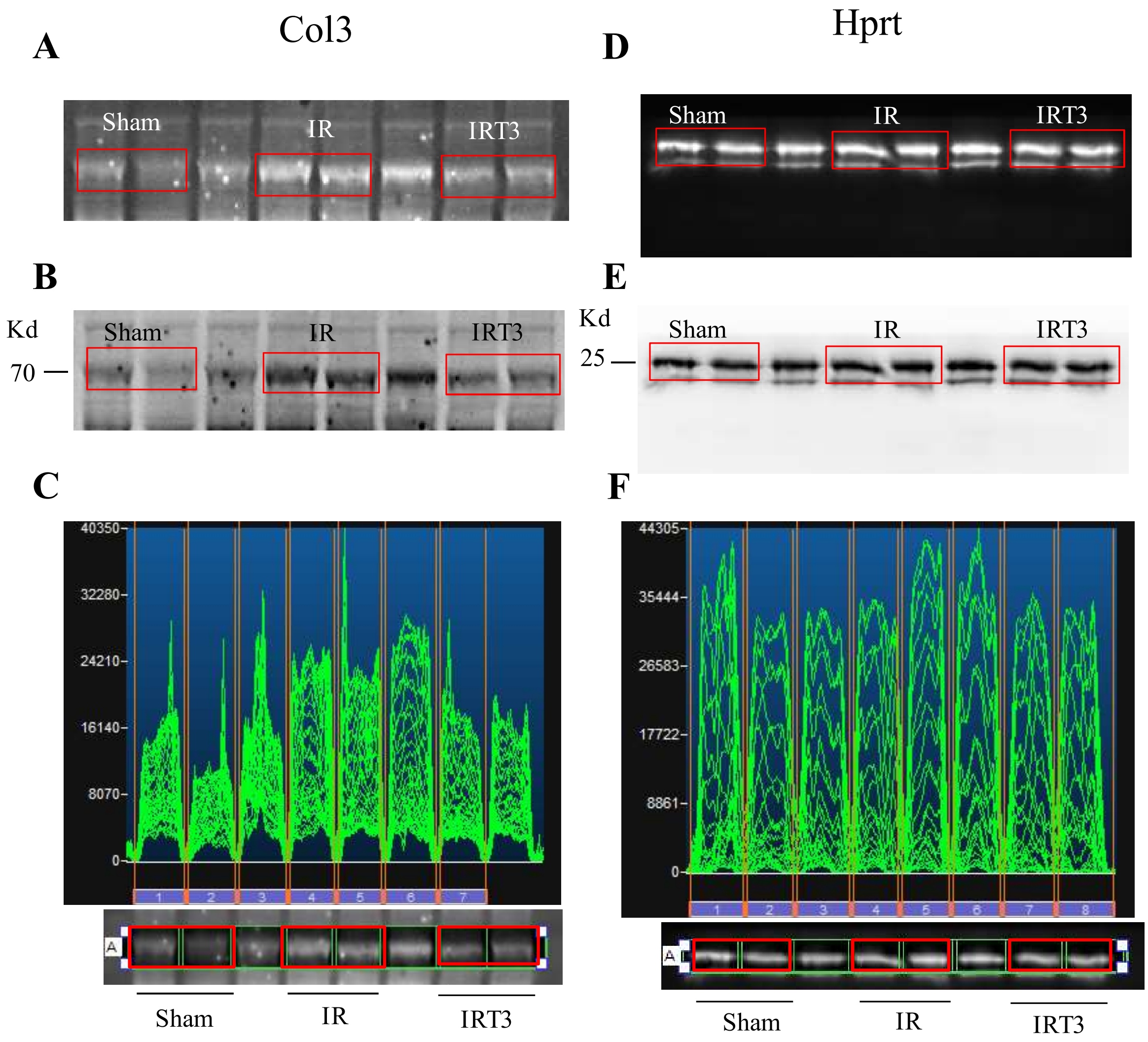

Supplementary Figure S4. Col3 protein level evaluation. **A.** Unprocessed Mmp2 specific chemiluminescent signal as acquired with the camera of the Alliance Mini2 Chemiluminescence Documentation System (UVITEC Cambridge). B. The original image was inverted for generating the cropped Figure 1D shown in the main text. C. The unprocessed original image was used to quantify the optical density through the ALLIANCE-CAPT Advance Software (UVITEC Cambridge) after excluding signal saturation as evidenced in the upper plot of the panel C. The bands of interest used in the cropped Figure 2D are marked with the red rectangles. C, D and E: Hprt specific chemiluminescent signal used for normalization was acquired and analyzed as described for Col3.

Supplementary table S1. List of genes assessed with the mitochondria and fibrosis PCR profiler arrays (MPA and FPA) grouped according to the functional category provided by the manufacturer

MITOCHONDRIA ARRAY	
**Apoptosis**	Aifm2, Akt1, Bak1, Bbc3, Bcl2, Bcl2l1, Bid, Bnip3, Cdkn2a, Gpx1, Pmaip1, Sfn, Sh3glb1, Sod2, Tp53 .
**Membrane Polarization**	Bak1, Bcl2, Bcl2l1, Bnip3, Gclc, Gclm, Sod1, Tp53, Ucp1, Ucp2, Ucp3
**Mitochondrial Transport**	Aip, Bak1, Bcl2, Bcl2l1, Bnip3, Cpt1b, Cpt2, Fxc1 (Timm10b), Grpel1, Hsp90aa1, Hspd1, Mfn2, Mipep, Mtx2, Stard3, Tp53, Tspo, Ucp1, Ucp2, Ucp3.
**Small Molecule Transport**	Slc25a10, Slc25a12, Slc25a13, Slc25a14, Slc25a15, Slc25a16, Slc25a17, Slc25a19, Slc25a2, Slc25a20, Slc25a21, Slc25a22, Slc25a23, Slc25a24, Slc25a25, Slc25a27, Slc25a3, Slc25a30, Slc25a36, Slc25a37, Slc25a4, Slc25a5
**Targeting Proteins to Mitochondria**	Aip, Timm10b (Timm10b), Grpel1, Hspd1, Mfn2, Mipep
**Mitochondrion Protein Import**	Aip, Akt1, Cav2, Cln8, Cox18, Timm10b (Timm10b), Gpx1, Grpel1, Hspd1, LOC691853 (Cox10), Mipep, Ppargc1a, Sh3glb1, Rnf135
**Outer Membrane Translocation**	Tomm22, Tomm34, Tomm40, Tomm40b, Tomm70a
**Inner Membrane Translocation**	Timm10b (Timm10b), Immp1l, Opa1, Taz, Timm10, Timm17a, Timm17b, Timm22, Timm44, Timm8a1, Timm8b, Timm9.
**Mitochondrial Fission and Fusion**	Cox18, Fis1, LOC691853 (Cox10), Dnm1l, Mfn1, Mfn2, Opa1

FIBROSIS ARRAY	
**ECM Components**	Col1a2, Col3a1
**ECM Remodeling Enzymes**	Mmp14, Mmp2 (Gelatinase A), Mmp8, Mmp9 (Gelatinase B), Plat (tPA), Plg, Serpin a1 (a1-antitrypsin), Serpine1 (PAI-1), Serpin h1, Timp1, Timp2, Timp3, Timp4.
**Cellular Adhesion**	Itga1, Itga2, Itga3, Itgav, Itgb1, Itgb3, Itgb5, Itgb6, Itgb8
**Inflammatory Cytokines and Chemokines**	Ccl11 (Eotaxin), Ccl12, Ccl3 (MIP-1a), Cxcr4, Ifng, Il10, Il1a, Il1b, Ilk, Tnf, Faslg
**Growth Factors**	Agt, Ctgf, Edn1, Hgf, Vegfa
**TGFß Superfamily**	Bmp7, Cav1, Dcn, Eng (EVI-1), Inhbe, Ltbp1, Smad2, Smad4, Smad6, Smad7, Tgfb1, Tgfb2, Tgfb3, Tgfbr1 (ALK5), Tgfbr2, Tgif1, Thbs1, Thbs2
**Transcription Factor**	Cebpb, Jun, Myc, Nfkb1, Sp1, Stat1, Stat6, Tbp
**Epithelial-to-Mesenchymal Transition**	Akt1, Bmp7, Col1a2, Col3a1, Ilk, Itgav, Itgb1, Mmp2 (Gelatinase A), Mmp3, Mmp9, Serpine1 (PAI-1), Smad2, Snai1 (Snail), Tgfb1, Tgfb2, Tgfb3, Timp1

Supplementary table S2 . List of differentially expressed miRNAs 3d following IR in the absence and presence of 48h T3 treatment

miR-ID	IR	IRT3	miR-ID	IR	IRT3
Down-regulated by ischemia			*Up-regulated by ischemia*		
miR-195-5p	0.50	0.53	**miR-222-3p**	**25.23**	**13.82#**
miR-30a-3p	0.50	0.64	**miR-31a-5p**	**17.75**	**10.33#**
miR-3068-3p	0.48	0.53	miR-223-3p	16.51	12.93
miR-30b-5p	0.48	0.66	miR-188-5p	15.52	10.09
miR-30d-5p	0.47	0.51	miR-21-5p	15.11	11.04
miR-181a-2-3p	0.47	0.54	miR-130b-3p	14.12	10.27
miR-125b-5p	0.47	0.59	miR-21-3p	13.58	11.24
miR-26a-5p	0.47	0.68	miR-34c-5p	13.09	10.55
miR-125b-5p	0.46	0.59	miR-484	12.80	11.38
miR-30e-3p	0.46	0.59	miR-142-3p	9.5	7.03
miR-22-3p	0.44	0.54	miR-214-3p	7.0	5.88
miR-26b-5p	0.43	0.57	miR-142-5p	6.0	5.10
miR-30c-5p	0.43	0.58	miR-221-3p	6.42	5.60
miR-139-5p	0.43	0.60	miR-146b-5p	6.34	6.37
miR-23a-3p	0.42	0.48	**miR-155-5p**	**6.28**	**3.74#**
miR-201-5p	0.42	0.65	miR-125b-1-3p	6.10	4.38
rmiR-1843-5p	0.42	0.48	miR-152-5p	4.82	3.35
miR-192-5p	0.41	0.52	miR-298-5p	4.47	2.95
miR-3585-5p	0.40	0.46	miR-27a-5p	4.41	2.36
miR-29a-3p	0.39	0.63	miR-193-3p	4.32	5.05
miR-10a-5p	0.39	0.49	miR-144-5p	4.12	6.16
miR-127-3p	0.39	0.36	miR-15b-5p	4.11	2.94
miR-24-3p	0.39	0.61	**miR-144-3p**	**3.54**	**6.80#**
miR-181d-5p	0.37	0.48	miR-582-3p	3.51	2.09
miR-361-5p	0.36	0.40	miR-340-5p	3.35	2.42
miR-9a-5p	0.36	0.45	miR-132-3p	3.27	2.28
miR-23b-3p	0.36	0.43	miR-92a-3p	3.27	2.38
miR-331-3p	0.34	0.62	miR-421-3p	3.26	2.19
miR-490-3p	0.34	0.48	miR-872-5p	3.19	2.48
miR-99a-5p	0.34	0.43	miR-199a-3p	2.82	2.19
miR-145-3p	0.34	0.47	miR-106b-3p	2.75	2.18
miR-378b	0.34	0.33	miR-19a-3p	2.72	2.15
miR-145-5p	0.34	0.51	miR-423-5p	2.62	1.77
miR-181c-3p	0.33	0.38	miR-17-5p	2.60	2.36
miR-378a-5p	0.32	0.42	miR-17-5p	2.40	2.23
rmiR-101a-3p	0.32	0.42	miR-423-3p	2.31	1.82
miR-99a-3p	0.31	0.37	miR-351-5p	2.21	1.51
miR-30a-5p	0.30	0.39	miR-92a-3p	2.19	1.77
miR-185-5p	0.29	0.48	miR-199a-5p	2.12	1.80
miR-181c-5p	0.28	0.33	miR-872-3p	2.10	2.31
miR-100-5p	0.28	0.40	miR-27b-5p	2.09	1.54
miR-208a-3p	0.28	0.44	miR-148b-5p	2.09	1.83
miR-22-5p	0.28	0.52	**miR-451-5p**	**1.43**	**3.44#**
miR-190a-5p	0.27	0.43			
miR-133a-5p	0.26	0.42			
**miR-29c-3p**	**0.25**	**0.49#**			
miR-30e-5p	0.25	0.35			
miR-10b-5p	0.25	0.38			
miR-345-5p	0.24	0.35			
**miR-208b-3p**	**0.23**	**0.41#**			
**miR-133a-3p**	**0.23**	**0.38#**			

miR-29b-3p	0.23	0.38
miR-29b-3p	0.22	0.38
**miR-338-3p**	**0.22**	**0.45#**
**miR-1-3p**	**0.21**	**0.38#**
miR-204-5p	0.20	0.23
**miR-208a-5p**	**0.19**	**0.44#**
**miR-499-5p**	**0.17**	**0.31#**
**miR-133b-3p**	**0.16**	**0.30#**

Values are mean fold changes relative to Sham control. miRNAs significantly affected by T3 vs IR (T3DE-miRNAs) are evidenced in bold. Adjusted p value vs Sham ≤0.02 for both IR and IRT3 rats, #adjusted p value IRT3 vs IR ≤ 0.04.

Supplementary table S3. MiRWalk significantly enriched miRNAs on KEGG pathways selected using the terms emerged from the functional enrichment analysis of the T3 differentially expressed genes (T3DE-genes).

**Targeted pathways**	**Enriched miRNAs in the selected pathways**													
	**133a-3p**	**133b-3p**	**1-3p**	**208a-5p**	**208b-3p**	**499-5p**	**338-3p**	**29c-3p**	**30-fam.**	**451a-5p**	**144-3p**	**222-3p**	**31a-3p**	**155-5p**
**Apoptosis**	X	X	X	X	X	X		X	X		X	X	X	X
**p53 signaling pathways**	X	X	X	X	X	X		X	X		X	X	X	X
**Tgfb signaling**	X	X		X	X	X	X	X	X	X	X			
**ECM receptor pathways**	X	X	X	X	X	X	X	X	X	X	X	X	X	X
**Hypertrophic cardiomyopathy**	X	X	X	X	X	X	X	X	X	X	X	X	X	X
**Dilated cardiomyopathy**	X	X	X	X	X	X	X	X	X		X	X	X	X

Supplementary table S4. List of the primers used in the qRT-PCR experiments

Gene	Sequence	Accession number
Bax	F 5'-CCCAGCGTCGTGATTAGTGATG-3'	NM_017059.2
	R 5'- GTGGGGGTCCCGAAGTAG-3'	
Bnip3	F 5'-GCGCAGCATGAATCTGGACG-3'	NM_053420.3
	R 5'-TGGTGTCTGGGAGCGAGGTG-3'	
Col3	F 5' TCCCCTGGAATCTGTGAATC -3'	NM_032085.1
	R 5'-TGAGTCGAATTGGGAGAAT-3'	
Cpt2	F 5'-CCCACCATGCACTACCAGGA-3'	NM_012930.1
	R 5'-GTATCTCTTCATGGTGTCTTCAAG-3'	
Ctgf	F 5'- GCTGACCTAGAGGAAAACATTAAGA-3'	NM_022266.2
	R 5'- CCGGTAGGTCTTGACACTGG-3'	
Itgb1	F 5'- CCAATCTTCCATGATGCACTTA-3'	NM_017022.2
	R 5'- CAGAGGTTTTTGCTCCTGAAGT-3'	
Mfn2	F 5'-CTGCAGCCACCAAGTTCAGCA-3'	NM_130894.4
	R 5'-TTTCTTGTTCATGGCAGCAA-3'	
p53	F 5'- AGAGAGCACTGCCCACCA-3'	NM_030989
	R 5'- AACATCTCGAAGCGCTCA-3'	
Ppargc1	F 5'- GCAATTTTTCAAGTCTAACTATGCAG-3'	NM_031347.1
	R 5'-AATCCAGAGAGTCATACTTGCTCT-3'	
Smad2	F 5'- CAAGGGTAACAATCCACACTC-3'	NM_019191.2
	R 5'- CGGAAGAGGAAGGAACAAAA-3'	
Sod2	F 5'- GGCCATATCAATCACAGCATT-3'	NM_017051.2
	R 5'- TAGCCTCCAGCAACTCTCCT-3'	
Slc25a20	F 5'-GTGTGCTTCTTTGGGTTTGG-3'	NM_053965.2
	R 5'-CCAGATAACATCCCAGCTGTAAA-3'	
Stat1	F 5'- CAGATATTATTCGCAATTACAAAGTC-3'	NM_032612.3
	R 5' -GATACTTCAGGGGATTCTCTGGT-3'	
Timp2	F 5'- CGTTTTGCAATGCAGACGTA-3'	NM_021989.2
	R 5'-GATGGGGTTGCCATAGATGT-3'	
Tgfb1	F 5'-CCTGGAAAGGGCTCAACAC-3'	NM_021578.2
	R 5'-CCTGGAAAGGGCTCAACAC-3'	
Tgfb3	F 5'-GCGTGGACAATGAGGAT-3'	NM_013174.2
	R 5'-GCAGTTCTCCTCCAAGTTGC-3'	
Tgfbr1	F 5'- TGCCTGCTTCTCATCGTGT-3'	NM_019191.2
	R 5'-AGGTGGCAGAAACACTGTAATG-3'	
Hprt	F 5'-CCCAGCGTCGTGATTAGTGATG-3'	NM_012583.2
	R 5'- ACTCTTCATTCAGGCCCTTG-3'	

miRNA	Sequence	Accession number
222-3p	5'-AGCTACATCTGGCTACTGGGT-3'	MIMAT0000891
31a-5p	5'-AGGCAAGATGCTGGCATAGC-3'	MIMAT0000810

155-5p	5'-TTAATGCTAATTGTGATAGGGGT-3'	MIMAT0030409
144-3p	5'-TACAGTATAGATGATGTACT-3'	MIMAT0000850
451-5p	5'-AAACCGTTACCATTACTGAGTT-3'	MIMAT0001633
29c-3p	5'-TAGCACCATTTGAAATCGGTTA-3'	MIMAT0000803
208b-3p	5'-ATAAGACGAACAAAAGGT-3'	MIMAT0017845
208a-5p	5'-GAGCTTTTGGCCCGGGTTATAC-3'	MIMAT0017844
499-5p	5’-TTAAGACTTGCAGTGATGTTT--3'	MIMAT0003381
133a-3p	5'-TTTGGTCCCCTTCAACAAGCTG-3'	MIMAT0000839
133b-3p	5'-TTTGGTCCCCTTCAACCAGCTA-3'	MIMAT0003126
1-3p	5'-TGGAATGTAAAGAAGTGTGTAT-3'	MIMAT0003125
338-3p	5'-TCCAGCATCAGTGATTTTGTTG-3	MIMAT0000581
30c	5'-TGTAAACATCCTACACTCTCAGC-3'	MIMAT0000804
SnRNA-U1	5'-CGACTGCATAATTTGTGGTAG-3'	
SNORA-55	5'-AGCCAACCTTGGAGAGCTGAGC-3’	
Universal primer 1	5'-TGAATCGAGCACCAGTTACGC-3'	
